# Guidance for Canadian Breast Cancer Practice: National Consensus Recommendations for Clinical Staging of Patients Newly Diagnosed with Breast Cancer

**DOI:** 10.3390/curroncol31110533

**Published:** 2024-11-15

**Authors:** Jeffrey Q. Cao, Brae Surgeoner, Mita Manna, Jean-François Boileau, Karen A. Gelmon, Muriel Brackstone, Christine Brezden-Masley, Katarzyna J. Jerzak, Ipshita Prakash, Sandeep Sehdev, Stephanie M. Wong, Nathaniel Bouganim, David W. Cescon, Stephen Chia, Ian S. Dayes, Anil Abraham Joy, Jan-Willem Henning

**Affiliations:** 1 Arthur Child Comprehensive Cancer Centre, Calgary, AB T2N 5G2, Canada; 2 Alberta Health Service, Calgary, AB T2S 3C3, Canada; 3 Saskatoon Cancer Centre, Saskatoon, SK S7N 4H4, Canada; 4 Jewish General Hospital, Montreal, QC H3T 1E2, Canada; 5 Department of Medical Oncology, University of British Columbia, Vancouver, BC V5Z 1M9, Canada; 6 London Health Sciences Centre, London, ON N6A 5W9, Canada; 7 Mount Sinai Hospital, Toronto, ON M5G 1X5, Canada; 8 Sunnybrook Health Sciences, Toronto, ON M4N 3M5, Canada; 9 The Ottawa Hospital Cancer Centre, Ottawa, ON K1H 8L6, Canada; 10 McGill University Health Centre, Montreal, QC H4A 3J1, Canada; 11 Princess Margaret Cancer Centre, Toronto, ON M5G 2M9, Canada; 12 BC Cancer—Vancouver, Vancouver, BC V5Z 4E6, Canada; 13 Juravinski Cancer Center, McMaster University, Hamilton, ON L8V 5C2, Canada; 14 Cross Cancer Institute, Edmonton, AB T6G 1Z2, Canada

**Keywords:** breast cancer, staging, recommendations, REAL Alliance

## Abstract

The accurate staging of breast cancer is fundamental for guiding treatment decisions and predicting patient outcomes. However, there can be considerable variation in routine clinical practice based on individual interpretation of guidelines and depending on the healthcare provider initially involved in working up patients newly diagnosed with breast cancer, ranging from primary care providers, triage nurses, surgeons, and/or oncologists. The optimal approach for clinical staging, particularly in asymptomatic patients presenting with intermediate-risk disease, remains a topic of dialogue among clinicians. Given this area of uncertainty, the Research Excellence, Active Leadership (REAL) Canadian Breast Cancer Alliance conducted a modified Delphi process to assess the level of agreement among Canadian expert clinicians on various staging recommendations. In total, 20 items were drafted covering staging based on biological status, the utilization of localization clips, both for the axilla during diagnosis and primary surgical site for margins and radiation therapy planning, and the use of advanced imaging for the investigation of distant metastases. Overall, the consensus threshold among all participants (i.e., ≥75% agreement) was reached in 20/20 items. Differences in clinical practice and recent findings from the literature are provided in the discussion. These consensus recommendations are meant to help standardize breast cancer staging practices in Canada, ensuring accurate diagnosis and optimal treatment planning.

## 1. Introduction

Research Excellence, Active Leadership (REAL) Canadian Breast Cancer Alliance is a standing nucleus committee of clinical-academic oncologists across Canada and the patient advocacy organization for Breast Cancer Canada (BCC) [1,2]. It was formed and launched in December 2023 in recognition that a national collaboration of leadership is needed to address an unmet need: to provide evidence-based guidance and recommendations, with timely updates, for use with public and government stakeholders for equitable and timely access and care for persons with breast cancer. This paper, aligned with REAL Alliance’s mission, seeks to unify patients, health policymakers, and oncologists in a collaborative effort to enhance breast cancer outcomes nationwide. It focuses on staging practices and is part of a series of recommendation papers released this year, which will be regularly updated as new evidence emerges.

Despite advances in early detection and treatment, the accurate staging of breast cancer remains a cornerstone of effective clinical management, guiding treatment decisions and aiding prognosis. However, across Canada, there can be significant variation in the application of staging guidelines, regional healthcare policies, resource availability, and interpretation of clinical evidence. This results in divergent treatment approaches and potential inequalities in patient care [3,4].

The challenge of harmonizing staging practices is noteworthy in early-stage breast cancer, particularly in human epidermal growth factor receptor 2 (HER2+) or triple-negative disease (TNBC). This is due to the increasing use of neoadjuvant systemic treatment. Historically, chemotherapy and staging were predominantly used in more locally advanced diseases. For example, Choosing Wisely, in collaboration with the American Society of Clinical Oncology (ASCO), highlighted investigations whose common use and clinical value were not supported by evidence. The release from 4 April 2012 included “Don’t perform positron emission tomography (PET), computed tomography (CT), and radionuclide bone scans in the staging of early breast cancer at low risk for metastasis” and also “Don’t perform surveillance testing (biomarkers) or imaging (PET, CT, and radionuclide bone scans) for asymptomatic individuals who have been treated for breast cancer with curative intent” [5]. Oncologists now must consider a complex landscape of clinical presentations, including the integration of biological markers, the use of localization clips and/or markers at the time of diagnosis and during treatment, and the use of advanced imaging techniques for detecting distant metastases. The issue with staging is further complicated by the lack of level 1 evidence with limited randomized controlled trials to clearly guide investigations and judicious allocation of limited resources. Considering these challenges, REAL Canadian Breast Cancer Alliance conducted a modified Delphi process to assess the level of agreement among expert clinicians on key staging recommendations for persons newly diagnosed with breast cancer.

## 2. Materials and Methods

A systematic review of the literature on advanced imaging in breast cancer staging was conducted by Alberta’s Guideline Resource Unit (GURU) (see Supplementary Tables S1–S3 and Supplementary Figure S1) and used by a sub-committee to develop a 33-item online survey (see Supplementary Table S4). This survey was designed to identify key areas of concern in breast cancer staging in Canada. The survey and the literature review were then distributed via an electronic platform to an expert panel, which included 12 medical oncologists, two surgical oncologists, two radiation oncologists, and a representative from BCC for the patient perspective. Panellists voted anonymously and indicated their level of agreement (Agree with statement as is, Agree with statement with edits, Do not agree with statement, or Abstain), suggested revisions, and commented on specific references and background data. All medical experts were affiliated with various academic institutions and professional societies across Canada, with specialized expertise and extensive experience in breast cancer management.

In June 2024, the group gathered for a two-day consensus conference in Toronto, Ontario, Canada, where the survey results were presented and discussed. During this meeting, with nine experts from the panel in attendance, 20 recommendations for breast cancer staging were drafted. These recommendations addressed staging based on biological status, the use of localization clips/markers and the use of advanced imaging for investigating distant metastases. The 20 recommendations as well as a summary of the meeting discussion were then sent to the full panel and subjected to anonymous voting as part of a modified Delphi process. Statements that did not achieve ≥ 75% agreement were revised and sent for a second round of voting. Consensus was reached on all 20 recommendations after two rounds of anonymous voting (Supplementary Table S5). The strength of each recommendation was determined based on the guidance from the GRADE system for evidence quality assessment and strength-of-evidence [6]. A strong recommendation is based on evidence from a randomized, controlled trial in the intention-to-treat population and is considered standard of care. A strong consideration is based on a sub-population from a randomized controlled trial. A moderate recommendation is based on lower levels of evidence. Expert opinion is offered in situations where a clinical decision is required but there is limited evidence.

These recommendations for staging investigations are in addition to comprehensive medical and family history assessments including menopausal status, directed physical examination of the patient with permission and offer of chaperone as well as routine bloodwork prior to surgery and systemic and/or radiation therapy if indicated. These recommendations were not focused on special populations or considerations such as patients with Li–Fraumeni syndrome or pregnancy nor distinct histopathologic types of cancer such as breast sarcomas, phyllodes tumours, or breast lymphomas that fall under soft tissue sarcomas and hematological malignancies.

## 3. Results

### 3.1. Anatomic Stage Group as Guide for Additional Staging Investigations

Overuse of staging investigations can lead to unnecessary resource consumption, increased healthcare costs, patient anxiety, psychological distress, and treatment delays. Conversely, failing to detect distant metastases during the initial workup may result in unwarranted treatments, including inappropriate surgeries and radiation therapy. Most international guidelines concur that additional imaging for distant metastases during workup is generally unwarranted in asymptomatic patients with early-stage breast cancer (stage I–II) [7,8,9,10]. However, there is significant variability in recommendations regarding which risk factors might justify further imaging. For example, the National Comprehensive Cancer Network (NCCN) notes routine systemic staging is not indicated in the absence of signs or symptoms of metastatic disease, but rather considered “for patients who are clinically high risk”, or prior to preoperative systemic therapy and can include T1cN0 for HER2-positive or TNBC [7]. Whereas European guidelines recommend staging investigations for cN+ and large tumours (>5 cm), and less specifically for “aggressive biology and in clinical signs, symptoms or laboratory values suggesting the presence of metastases” [8]. This variability in recommendation is also observed within Canada, where there is no national consensus as to what should guide additional staging investigations. In Quebec [11] and British Columbia [12] the decision to order additional investigation can be swayed by the biomarker status given the poorer prognosis associated with HER2+ or TNBC cancers. In Alberta and Ontario, additional investigation is not recommended for stage I–II regardless of biomarker status [9].

Despite the increased recurrence risk associated with certain biological subtypes, evidence does not consistently show a direct correlation between specific breast cancer subtypes and the onset of metastasis [13,14,15]. Studies using advanced imaging like PET-CT indicate that detection rates are more closely tied to the breast cancer stage rather than the biological subtype, with stage III showing the highest rates across major subtypes. A literature review conducted by Cancer Care Ontario (CCO) [9] showed that PET-CT on stage III breast cancer had distant metastasis detection rates of 26%, 22%, and 32% across estrogen receptor-positive (ER+), HER2+ and TNBC subtypes respectively, whereas for stage II the detection rates were consistent at 10% across subtypes. In contrast, stage I had lower detection rates, such as 7% for ER+ and 0% for both HER2+ and TNBC. With conventional imaging, similarly low detection rates were observed across these subgroups in stage I-II of the disease. It should be noted that slightly higher detection rates with abdominal CT in HER2+ patients have also been reported, however, while this result suggests that additional staging investigations should be warranted for this subgroup of breast cancer, these findings were not stratified by clinical stage [16]. In contrast to molecular subtype, there is a clear correlation between tumour size and the likelihood of metastasis, particularly in the 1.0 to 5.0 cm range [17,18,19]. The same holds true for nodal status. A retrospective study of 370 women found metastasis upstaging rates of 8.1%, 12.8%, 24.5%, and 38.7% for cT1 to cT4 tumour size, and 5.7%, 22.4%, 48.1%, and 80.0% for cN0 to cN3 nodal involvement, respectively [20]. Based on this evidence, and how the presence of nodal disease is generally regarded as the highest risk factor for distant metastasis and an indicator for adjuvant therapies, REAL Alliance concluded, with 94% agreement, that additional imaging investigations for distant metastasis should be guided by the size, nodal and metastatic status (TNM) rather than the biological subtype of breast cancer (Table 1).

REAL Alliance also evaluated whether the decision to perform advanced imaging should be influenced by the need for neoadjuvant therapy. Traditionally, staging has been recommended for locally advanced disease when neoadjuvant chemotherapy is planned. With the increasing use of neoadjuvant systemic therapy in earlier-stage cases, the appropriate timing for staging investigations has become less clear. In current practice for TNBC and HER2+ early breast cancer, neoadjuvant therapy is commonly used. While clinical trials often include extensive staging in these populations, it remains uncertain whether this approach should be routinely adopted in real-world settings. The challenge lies in the risk of identifying distant metastases in patients eligible for neoadjuvant therapy, which could result in upstaging and exclusion from potentially curative treatments. St. Gallen’s 2021 recommendations state that “when patients are planned to receive neoadjuvant systemic therapy, in general [staging investigation] should be considered as a standard evaluation” [21]. This topic was the most divisive among REAL members, as while investigation for distant metastases is not recommended based on cancer subtype, some highlighted how clinical staging is not always accurate, particularly for the axilla [22]. Despite this, the majority (75%) of REAL Alliance panel voted against using the neoadjuvant or adjuvant setting as a basis for deciding whether to order additional imaging for distant metastases (Table 1). 

### 3.2. Magnetic Resonance Imaging (MRI) for Local and Regional Workup

The role of MRI in breast cancer diagnostics is a topic of ongoing dialogue. For specific scenarios, such as identifying clinically occult primary tumours presenting with axillary nodal metastases or assessing Paget’s disease when mammography is inconclusive, MRI can be a valuable tool [23,24,25]. It is also useful for screening patients with a high risk of breast cancer based on family history [26]. However, most guidelines do not universally recommend MRI, considering it optional and situation-dependent, with its use being guided by specific clinical indications rather than as a routine procedure [7,8,27].

MRI has exceptional sensitivity in detecting the extent of disease, especially in dense breast tissue where traditional imaging methods like mammography may fail to identify hidden malignancies. MRI is particularly effective in evaluating invasive cancers and guiding surgical decisions by providing detailed information on the extent of the tumour. However, this high sensitivity can also be a drawback as MRI may lead to false positives. This results in additional diagnostic procedures, such as MRI-guided biopsies, and may contribute to an increased rate of mastectomies due to overestimation of disease extent [28,29,30,31,32].

The REAL panel voted (87%) to align with Cancer Care Ontario (CCO) guidelines regarding recommendations on imaging for local and regional workups and the use of MRI (Table 2). The panel, however, added that MRI is also warranted for the initial workup in breast cancer patients with a history of mantle field radiation or thoracic irradiation to assist in differentiating between malignancy and radiation-induced changes such as fibrosis.

### 3.3. Localization Prior to Surgery

It is considered standard of care for a biopsy clip to be placed in the breast lesion at the time of diagnostic biopsy for future localization [7,33]. However, the benefit of clipping axillary nodes for restaging after neoadjuvant chemotherapy (NAC) remains subject for debate. Accurate axillary staging (an integral part of establishing pathological complete response (pCR) status) is important to optimize adjuvant systemic therapy and radiotherapy treatment recommendations [34,35,36,37]. Postoperatively, guidelines agree that placing surgical clips in the primary site of resection is useful to delineate the tumour bed for radiation planning, including the design of boost and accelerated partial breast irradiation (APBI) fields, and to mark the site if further surgery is needed based on margin status [7,8,33]. 

To put this into context, axillary lymph node dissection (ALND) has traditionally been the standard treatment for clinically node-positive patients but is associated with significant morbidity. Its necessity has been put into question particularly because systemic NAC can result in a nodal pCR [38]. To reassess the value of ALND after NAC, some trials have explored using sentinel lymph node biopsy (SLNB) to re-evaluate nodal status for de-escalation of treatment [39]. Based on this and other trials, it has become clear that performing an SLNB can safely restage the axilla when a dual tracer is utilized to increase the yield of identified sentinel nodes to at least three in patients who are clinically node-negative following the completion of NAC [33,39,40,41,42]. Notably, some studies have proposed that placing this clip in a biopsy-confirmed metastatic node and targeting the removal of the marked node at the time of surgery (targeted axillary dissection (TAD)) can reduce its false negative rate [39,43]. For instance, in 2016 Caudle et al. demonstrated that clipping nodes at diagnosis reduced the false negative rate from 14% to 2% during restaging, compared to 4% when only the clipped node was removed [43]. Similarly, the prospective multi-center RISAS trial showed a false negative rate of 3.5% in patients who had pre-operative node clipping as compared to 17.9% with SLNB alone [44]. Although the small sample sizes from these individual studies are a limitation, a recent meta-analysis has confirmed these promising findings, suggesting that clipping may help reduce false negative rates and avoid unnecessary ALND and its associated morbidity [45]. Despite this, most current clinical guidelines do not require the placement of an axillary node clip in patients undergoing NAC. While REAL panel agrees (94%) with the potential benefits of clipping the axillary node, they acknowledge that it is based on weak evidence and that the decision to place markers for axillary nodes should be made in consultation with the breast surgeon and in accordance with local institutional practices (Table 3).

### 3.4. Imaging for Distant Metastases

#### 3.4.1. Conventional Imaging

Breast cancer commonly metastasizes to the bones, lungs, liver, pelvis, and brain [46]. Each site of metastasis may present with distinct clinical symptoms, which can guide the choice of conventional anatomic (chest X-ray (CXR), liver ultrasound, CT of chest/abdomen/pelvis) and/or metabolic imaging modalities (PET-CT, MRI) for detection and monitoring.

European guidelines state that the minimum imaging workup for staging includes CT of the chest/abdomen and bone scan [8]. This combination allows for a comprehensive evaluation of the most common metastatic sites in breast cancer. However, the interpretation of CT and bone scan must be cautious to avoid misdiagnosis due to the high rate of false positives [47,48,49]. Because of the lack of evidence supporting the use of these imaging modalities in the lower stages of breast cancer, CCO guidelines do not recommend staging investigation for distant metastasis in patients with Stage I or II [9]. European guidelines are more prudent with recommendations for Stage IIB and higher, clinically positive axillary nodes or “aggressive biology” [8]. Likewise, NCCN recommends staging investigations “only in patients who are clinically high-risk” [7]. In contrast, in the joint EANM-SNMMI guidelines, stage IIA is listed as optional for the investigation of distant metastases but acknowledges the lack of sufficient data for full support [50]. Considering this, REAL Alliance supports recommending these imaging modalities (100% for CT-chest/abdomen ± pelvis; 87% for bone scan) for patients with stages IIB-IIIC (not IIA), particularly when functional imaging such as PET-CT is not available (Table 4).

While CT-chest/abdomen ± pelvis and bone scan are preferred for breast cancer staging due to their higher sensitivity and specificity, CXR and liver ultrasound can still be useful in certain situations. CXR can be a quick and accessible option when patients have respiratory symptoms or when advanced imaging is not available, allowing for the detection of gross lung metastases. Similarly, liver ultrasound may be considered for patients who cannot undergo CT with contrast or in resource-limited settings. As a result, REAL panel agreed that CXR (94%) and liver ultrasound (87%) can be reasonable alternatives to CT-chest/abdomen ± pelvis (Table 4).

#### 3.4.2. PET/CT

PET-CT is increasingly being incorporated into staging investigations for detecting cancer cell activity due to its sensitivity in identifying distant metastases and extra-axillary lymph nodes compared to conventional imaging [51,52,53]. Considering that PET-CT is prone to false positives in cases of small tumours (≤2 cm, T1), it is not surprising to see that for early-stage ductal disease, particularly Stage I T1N0, PET-CT performs poorly [54,55]. A multicenter study of 325 women with operable stage I breast cancer found that PET-CT suggested distant metastases in 13 patients, but only 3 (0.9%) were confirmed as metastatic, with 10 (3%) being false positives [56]. In stage IIA, retrospective studies showed that detection rates with PET-CT were highly variable among studies, with results ranging from 0.9% to 11% [57,58,59]. Given this variability, it is unclear if PET-CT is warranted in this setting [50]. Conversely, PET-CT plays a more definitive role in stage IIB and higher, where the likelihood of detecting metastases increases. In the first and only randomized controlled trial questioning the performance of PET-CT compared to conventional imaging (OCOG PET ABC study), the Canadian group led by Dayes et al. showed that PET-CT identified distant metastases in 23% of stage IIB (T3N0, but not T2N1) and stage III breast cancer patients, compared to 11% with conventional imaging [60]. Similarly, PET-CT for baseline staging in stage IIB or higher has been shown to lower the false positive rate by 50% compared to conventional imaging [61]. PET-CT also can detect more extensive disease, including metastases in hard-to-reach nodal regions like the internal mammary and mediastinal lymph nodes [61,62,63,64]. Considering that PET-CT provides similar costs, a lower radiation dose than conventional imaging, and the convenience of completing a full investigation in a single visit, REAL Alliance unanimously agreed that PET-CT is an acceptable alternative to conventional imaging or if conventional imaging results are equivocal for patients with stage IIB-IIIC breast cancer (Table 4).

Despite the advantages of PET-CT, its use in the context of invasive lobular carcinomas (ILC) remains a subject of ongoing research [65]. ILC, which accounts for approximately 10–15% of breast cancer cases, poses unique challenges for PET-CT due to its lower uptake of the standard glucose radiotracers used for imaging, and its diffuse infiltrative growth pattern compared to non-lobular types. In a retrospective study, PET-CT was less effective at detecting unsuspected distant metastases in patients with stage III lobular disease (11%) versus those with ductal disease (22%) [66]. Despite this reduced sensitivity, PET-CT may still have potential value in ILC. A small retrospective study found that its performance was comparable to conventional imaging, even in non-glucose-avid tumors [67]. ILC is nearly always (95%) ER+, and therefore ER-targeting PET tracers such as 16α-18F-fluoroestradiol (18F-FES) are regarded as a solution to the glucose avidity issue [68]. However, current evidence remains insufficient to fully endorse PET-CT in ILC, and therefore REAL Alliance voted (92%) to withhold a stance for its routine use in this subtype (Table 4).

Another area of debate with PET-CT is its use in inflammatory breast cancer (IBC). IBC is the most aggressive form of breast cancer with approximately 80% of patients presenting with axillary lymph node involvement and 40% with distant metastases [69,70,71]. False positives in the context of a rapidly growing tumor can have paramount effects over the treatment decision as it can upstage patients prematurely and could result in patients being excluded from neo/adjuvant, curative intent therapies. In the OCOG PET ABC study, an exploratory subgroup analysis of patients with IBC did not reveal a difference in detection of distant metastases with PET-CT over conventional imaging. Of the limited sample size of 33 patients with IBC, 4/16 (25%) PET-CT patients were upstaged to Stage IV compared with 4/17 (24%) conventional patients [60]. Nevertheless, many guidelines recommend incorporating PET-CT in the staging of IBC given its higher sensitivity and specificity compared to conventional imaging (96–100% and 91–98% vs. 60–84% and 67–83% respectively) [72,73,74,75,76,77,78,79,80,81], as well as its high sensitivity for detection of breast lesions (96%) and axillary nodal involvement (95%) [73]. Given this information, REAL Alliance, with 93% agreement, voted that PET-CT may be recommended for staging patients with IBC (Table 4).

#### 3.4.3. Imaging of the Brain

Brain metastases (BM) are a significant cause of morbidity and mortality, particularly in patients with metastatic breast cancer with HER2+ and TNBC subtypes where the incidence rates are as high as 31% and 32%, respectively, compared to 15% in ER+ breast cancer [82]. This higher lifetime incidence is partly due to the inability of most chemotherapeutic agents and therapeutic antibodies to cross the blood-brain barrier in therapies for HER2+ and TNBC cases [83,84,85]. However, while the high rate of incidence of BM in the metastatic setting may warrant routine imaging of the brain, current evidence does not support imaging in the early setting in the absence of symptoms. A meta-analysis of the incidence of BM in non-metastatic breast cancer showed that, in this setting, the incidence of BM as the site of first recurrence per year of median follow-up ranged from 0.1% to 3.2% [86]. As such, international guidelines do not recommend routine CT or MRI scans for detecting brain metastases in asymptomatic breast cancer patients, regardless of subtype, due to the low likelihood of detection and the potential for unnecessary anxiety and interventions [7,8,86]. Brain imaging is typically reserved for patients who exhibit neurological symptoms or in cases of advanced disease with a strong suspicion of brain metastasis. Reflecting this, REAL Alliance, with 81% agreement, aligns with current international guidelines to not support routine brain imaging for staging asymptomatic patients, regardless of subtype (Table 4). 

### 3.5. Timing for Imaging Workup and Emerging Technologies

#### 3.5.1. Timing Consideration in Imaging Workup

The cancer workup process can be lengthy, often causing delays in treatment management and increasing patient anxiety. Although the Canada Health Act guarantees equitable access to high-quality care, delivered through provincial and territorial health care systems, significant delays persist across several provinces [87]. A study by the Fraser Institute in 2023 revealed that waiting times for imaging vary widely across Canada [88]. For example, the wait for a CT scan ranges from 4 weeks in Quebec to 14 weeks in Nova Scotia. Ultrasound waiting times span from 2 weeks in Saskatchewan to 14 weeks in Prince Edward Island, while MRI waits are the longest, ranging from 10 weeks in Quebec and Ontario to 25 weeks in Nova Scotia. Notably, these waiting times have increased compared to 2022 and 2021 data, underscoring the need for stricter policies and standards to ensure optimal care for breast cancer patients. In response, REAL Alliance unanimously endorsed (100%) the Canadian Association of Radiologists’ guideline benchmarks for wait times (Table 5) [89]. These guidelines recommend that investigative imaging be prioritized according to each case, with even the lowest priority cases being processed within a maximum of 60 days of referral. 

To address this challenge, breast health clinics (BHCs), established in Canada [90,91,92,93] are able to offer centralized breast cancer diagnostic and treatment services. These clinics have shown success in reducing wait times compared to standard care models. In Canada, BHCs are particularly valuable, especially in rural and remote areas, where they help alleviate the financial and emotional burdens associated with travelling for care [94].

#### 3.5.2. Alternative and Emerging Imaging Methods

As previously mentioned, novel ER-targeting radiotracers for PET are a particularly interesting imaging method not only for their use in ILC but also because this imaging modality provides valuable insights into the hormonal status of the tumour, which can guide treatment decisions, particularly in hormone-sensitive cancers [95]. Other emerging technologies are expanding the landscape of breast cancer imaging. Techniques such as contrast-enhanced mammography (CEM), automated breast ultrasound (ABUS), and advanced MRI protocols are offering enhanced sensitivity and specificity [96]. These innovations are not only improving early detection but are also crucial in monitoring treatment response and guiding therapeutic interventions. Recognizing the potential of these advancements, REAL Alliance (with 87% agreement), has emphasized the necessity of routinely reassessing and disseminating knowledge on emerging technologies in the field (Table 5). By integrating these tools into clinical practice once their validity and utility have been established, clinicians may improve the accuracy of breast cancer diagnoses and tailor treatments more effectively, ultimately enhancing the quality of care for breast cancer patients. 

## 4. Conclusions

**Figure 1:**
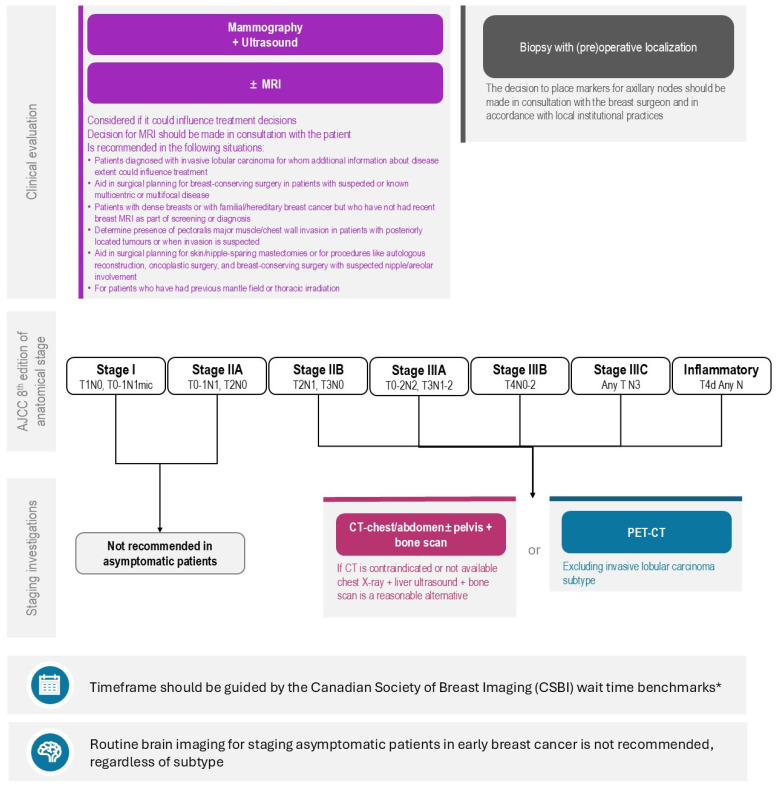
Algorithm for Staging Investigations for Patients Newly Diagnosed with Breast Cancer without Signs and/or Symptoms of Distant Metastases. REAL Alliance overall recommendation for imaging of distant metastasis based on anatomic stage of disease, local regional workup, and timing consideration for imaging workup. (*) Endorsed recommendation from the Canadian Association of Radiologists’ guideline benchmarks for benchmark wait times [89].

The accurate staging of breast cancer is essential for optimizing treatment decisions and improving patient outcomes. However, some variation in staging practices exists amongst practitioners, and across Canadian provinces, driven by differences in healthcare policies, resource availability, and clinical interpretations. This initiative, through a modified Delphi process, has highlighted areas of consensus and divergence among Canadian expert clinicians on key staging recommendations, particularly in the use of biological markers to inform the approach to staging, localization clips/markers, and advanced imaging techniques (Figure 1).

With consensus achieved on all of the final 20 items, the findings underscore the importance of developing more uniform staging guidelines across Canada. By addressing these potential disparities, REAL Alliance intends to enhance the consistency of breast cancer diagnosis and treatment planning, ensuring that all patients, regardless of location, receive the highest standard of care. The insights gained from this initiative are intended to guide future efforts in unifying breast cancer staging practices, ultimately contributing to better clinical outcomes and more equitable healthcare delivery across the country.

## Figures and Tables

**Table 1:** REAL Alliance recommendations for general considerations for staging along with strength of recommendation, level of agreement of the working group (%), and how they compare with international and provincial guidelines.

Recommendation	REAL	NCCN [7]	ESMO [8]	CCO [9]
For patients newly diagnosed with breast cancer and without signs and/or symptoms of distant disease, the decision to order staging investigations should be based on the Anatomic Stage Group as per the 8th Edition American Joint Committee on Cancer (AJCC) TNM Staging System.	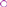 REAL Alliance Expert opinion 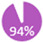	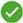	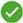	** 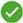 **
The decision to order staging investigations should not differ based on adjuvant or neoadjuvant approach nor should it be modified by subtype in either the neoadjuvant or post-operative settings.	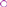 REAL Alliance Expert opinion 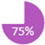	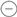 includes HER2+ and TNBC for T1cN0	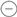 includes “aggressive biology”	** 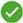 **

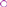
, REAL Alliance expert opinion; ●, Moderate recommendation; 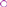

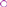
, Strong consideration; ●●, Strong recommendation. 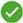, Alignment; 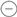, Some variation between guidelines; NC, not covered; NCCN, National Comprehensive Cancer Network; ESMO, European Society for Medical Oncology; CCO, Cancer Care Ontario.

**Table 2:** REAL Alliance recommendations for staging during local and regional workup along with strength of recommendation, level of agreement of the working group (%), and how they compare with international and provincial guidelines.

Recommendation	REAL	NCCN [7]	ESMO [8]	CCO [27]
Imaging for local and regional **workup with bilateral mammography as well as breast and axillary ultrasound is indicated** for all patients with suspected breast cancer.	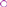 REAL Alliance Expert Opinion 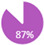	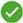	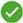	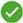
For **locally advanced disease (Stages IIB-IIIC), comprehensive imaging, including mammography, ultrasound, +/− MRI**, is indicated to evaluate the extent of local and regional disease.	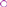 REAL Alliance Expert Opinion 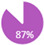	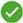	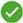	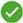
**Preoperative breast MRI should be considered** for patients diagnosed with breast cancer where additional information regarding **disease extent could influence treatment decisions.**	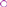 REAL Alliance Expert Opinion 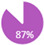	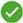	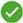	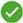
**Decision to conduct MRI should be made in consultation with the patient**, considering the balance of benefits and risks and patient preferences.	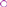 REAL Alliance Expert Opinion 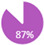	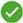	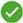	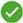
Preoperative breast MRI is recommended in the following situations: In patients diagnosed **with invasive lobular carcinoma** for whom additional information about disease extent could influence treatment To aid in surgical planning for breast-conserving surgery in patients with suspected or **known multicentric or multifocal disease.** To identify additional lesions in **patients with dense breasts** To determine the presence of pectoralis major muscle/chest wall invasion in patients with **posteriorly located tumours or when invasion is suspected**. To **aid in surgical planning for skin/nipple-sparing mastectomies or for procedures** like autologous reconstruction, oncoplastic surgery, and breast-conserving surgery with suspected nipple/areolar involvement. Patients **with familial/hereditary breast** cancer but who have not had recent breast MRI as part of screening or diagnosis	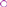 REAL Alliance Expert Opinion 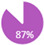	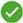	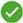 Includes presence of breast implants	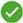
Preoperative breast MRI is recommended for patients who have **had previous mantle field or thoracic irradiation**.	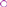 REAL Alliance Expert Opinion 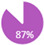	NC	NC	NC

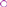
, REAL Alliance expert opinion; ●, Moderate recommendation; 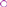

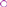
, Strong consideration; ●●, Strong recommendation. 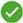, Alignment; 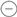, Some variation between guidelines; NC, not covered; NCCN, National Comprehensive Cancer Network; ESMO, European Society for Medical Oncology; CCO, Cancer Care Ontario.

**Table 3:** REAL Alliance recommendations for use of clips/markers during workup and treatment along with strength of recommendation, level of agreement of the working group (%), and how they compare with international and provincial guidelines.

Recommendation	REAL	NCCN [7]	ESMO [8]	CCO [33]
The placement of a marker is indicated at the time of core needle biopsy to mark the location of the primary tumour(s) and axillary node(s), especially if neoadjuvant therapy is planned. The decision to place markers for axillary nodes should be made in consultation with the breast surgeon and in accordance with local institutional practices.	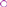 REAL Alliance Expert Opinion 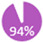	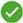	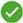	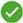
For breast conserving surgery and radiation therapy planning, **clips should be placed in the surgical bed**, especially if oncoplastic surgery is performed. This is useful to guide radiotherapy planning of boosts and allows for the option of accelerated partial breast irradiation.				

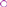
, REAL Alliance expert opinion; ●, Moderate recommendation; 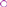

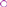
, Strong consideration; ●●, Strong recommendation. 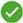, Alignment; 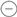, Some variation between guidelines; NC, not covered; NCCN, National Comprehensive Cancer Network; ESMO, European Society for Medical Oncology; CCO, Cancer Care Ontario.

**Table 4:** REAL Alliance recommendations for imaging of distant metastasis during staging workup along with strength of recommendation, level of agreement of the working group (%), and how they compare with international and provincial guidelines.

Recommendation	REAL	NCCN [7]	ESMO [8]	CCO [9]
Routine chest **X-rays are not indicated for staging but** may be used for initial evaluation in patients with respiratory symptoms or for baseline assessment in certain clinical scenarios.	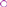 REAL Alliance Expert Opinion 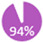	NC	NC	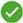
**CT Thorax/Abdomen ± Pelvis is indicated for patients with Stages IIB-IIIC**. Routine use in early-stage disease (Stages 0-IIA) is not recommended.	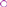 REAL Alliance Expert Opinion 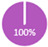	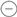 Only for patients who are clinically high risk	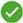 Includes clinically positive axillary nodes	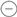 Stage III only
**Bone Scan is indicated for patients with Stages IIB-IIIC**. Routine use in early-stage disease (Stages 0-IIA) is not recommended.	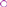 REAL Alliance Expert Opinion 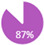	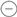 Only for patients who are clinically high risk	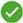 Includes clinically positive axillary nodes	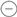 Stage III only
**Ultrasound of the liver is a reasonable alternative to CT abdomen if CT is contraindicated or not available.**	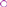 REAL Alliance Expert Opinion 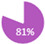	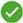	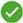	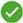
**PET-CT is an alternative, but not an additional staging investigation**, to conventional imaging with CT and bone scan in patients with **Stage IIB-IIIC** presentation.	● Moderate recommendation 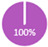	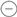 Only for patients who are clinically high risk	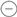 can be adjunct to diagnostic CT	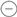 Stage III only
**PET-CT is not currently indicated for lobular breast cancer** (due to reduced sensitivity but is under further investigation).	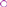 REAL Alliance Expert Opinion 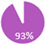	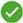	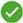	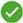
**PET-CT may be recommended for patients with inflammatory breast cancer.**	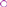 REAL Alliance Expert Opinion * 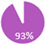 *	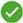	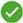	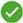
**PET-CT may be useful when conventional imaging results in equivocal findings.**	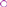 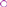 Strong consideration 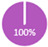	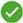	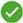	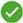
**CT-head or MR-brain is not routinely indicated** for staging asymptomatic patients, irrespective of subtype (i.e., HER2-positive and TNBC).	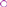 REAL Alliance Expert Opinion 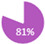	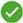	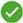	NC

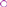
, REAL Alliance expert opinion; ●, Moderate recommendation; 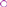

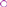
, Strong consideration; ●●, Strong recommendation. 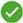, Alignment; 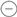, Some variation between guidelines; NC, Not covered; NCCN, National Comprehensive Cancer Network; ESMO, European Society for Medical Oncology; CCO, Cancer Care Ontario.

**Table 5:** REAL Alliance recommendations for timing of imaging and emerging technologies along with strength of recommendation, level of agreement of the working group (%), and how they compare with international and provincial guidelines.

Recommendation	REAL	NCCN	ESMO	CCO
Imaging workup should be **completed in a timeframe that will not impede the initiation or continuation of treatment**(s). Timeframe should be guided by the Canadian Society of Breast Imaging (CSBI) wait time benchmarks.	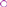 REAL Alliance Expert Opinion 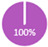	NC	NC	NC
Advanced PET technology, focusing on distinct metabolic processes in breast cancer imaging, demonstrates promise but is not currently routine standard of care. Reassessing and disseminating knowledge on these and other emerging technologies as evidence evolves is crucial to enhancing diagnostic accuracy and providing state-of-the-art care for breast cancer patients in Canada.	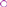 REAL Alliance Expert Opinion 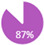	NC	NC	NC

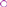
, REAL Alliance expert opinion; ●, Moderate recommendation; 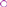

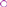
, Strong consideration; ●●, Strong recommendation. 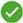, Alignment; 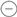, Some variation between guidelines; NC, Not covered; NCCN, National Comprehensive Cancer Network; ESMO, European Society for Medical Oncology; CCO, Cancer Care Ontario.

## Supplementary Materials

The following supporting information can be downloaded at https://www.mdpi.com/article/10.3390/curroncol31110533/s1, Figure S1: PRISMA flow diagram; Table S1: Summary of existing guideline recommendations used for drafting premeeting survey (published between April 2019–April 2024); Table S2: Summary of white literature used to draft premeeting survey (published between 2013–6 November 2023); Table S3: Literature search strategy; Table S4: Pre-meeting survey and voting results; Table S5: Expanded polling results for recommendations.
