# Supplementary material for: Guidance for Canadian Breast Cancer Practice: National Consensus Recommendations for Clinical Staging of Patients Newly Diagnosed with Breast Cancer
Source: Curr Oncol. 2024 Nov 15;31(11):7226–43. doi: 10.3390/curroncol31110533 (PMC11592626; doi:10.3390/curroncol31110533)
Supplement: Supplementary file 1 [file curroncol-31-00533-s001.zip › curroncol-3257633-supplementary.pdf]

Supplementary Table S1. Summary of existing guideline recommendations used for drafting premeeting survey (published between Apr 2019 – Apr 2024)

Guideline author, year	Stage	Staging Imaging for Asymptomatic Pts with Newly Diagnosed, Biopsy Proven BC
NCCN, 2024 (v2 –Mar. 11, 2024)	DCIS<br>Tis, N0, M0	• Breast MRI as indicated (use of MRI not shown to increase likelihood of negative margins or decrease conversion to mastectomy. Data to support improved long-term outcomes lacking)
	Localized BC: invasive, non-inflammatory, non-metastatic (M0)	• Breast MRI (optional), w special consideration for mammographically occult tumours (Breast MRI may be useful for characterizing axillary and/or internal mammary nodal disease)<br>• Consider additional imaging studies only in presence of signs and symptoms of metastatic disease (Routine systemic staging not indicated for non-metastatic [M0] cancer in absence of signs or symptoms)
	Prior to Preop Systemic Therapy<br><br>c≥T2 or cN+ and M0<br>**or**<br>cT1c, cN0 HER2+<br>**or**<br>cT1c, cN0 TNBC	Additional tests to consider as clinically indicated:<br>• Chest diagnostic CT +/- contrast<br>• Abdominal +/- pelvic diagnostic CT w contrast or MRI w contrast<br>• Bone scan or sodium fluoride PET/CT<br>• FDG PET/CT (most beneficial and accurate for advanced disease [stage III] and invasive ductal [compared to lobular] histology but may be useful in selected circumstances of earlier stage disease [stage IIA disease: T1N1, T2N0] such as: equivocal CT + bone scan results; suspicion of undetected nodal and/or distant disease; and treatment response assessment. FDG-PET/CT may be utilized as adjunct to, or in lieu of, initial standard staging and may be performed simultaneously w diagnostic CT. Conversely, bone scan or sodium fluoride PET/CT may not be needed if upfront FDG PET/CT clearly indicates consistent findings on both PET and CT components).<br>• Breast MRI (optional), w special consideration for mammographically occult tumours, if not previously done (Breast MRI may be useful for characterizing axillary and/or internal mammary nodal disease)
	Stage IV (M1)	• Chest diagnostic CT +/- contrast<br>• Abdomen +/- pelvis diagnostic CT w contrast or MRI w contrast<br>• Bone scan or sodium fluoride PET/CT<br>• Useful in certain circumstance:<br>o FDG-PET/CT (consider FES-PET/CT for ER+ disease)
	Principles of Dedicated Breast MRI Testing	**Clinical Indications and Applications**<br>• May be used for staging evaluation to define extent of cancer or presence of multifocal or multicentric cancer in ipsilateral breast, or as screening of contralateral BC at time of initial diagnosis (category 2B). No high-level data to demonstrate that use of MRI to facilitate local therapy decision-making improves local recurrence or survival.<br>• May be helpful for BC evaluation before and after preop systemic therapy to define extent of disease, response to treatment, and potential for breast-conservation therapy.<br>• May be useful in identifying otherwise clinically occult disease in pts presenting w axillary nodal mets (cT0, cN+), w Paget disease, or w invasive lobular carcinoma poorly (or inadequately) defined on mammography, US, or physical exam.

<table>
<tr><th>Guideline author, year</th><th>Stage</th><th>Staging Imaging for <u>Asymptomatic</u> Pts with Newly Diagnosed, Biopsy Proven BC</th></tr>
<tr><td></td><td></td><td>False-positive findings on breast MRI common. Surgical decisions should not be based solely on MRI findings. Additional tissue sampling of areas of concern identified by breast MRI is recommended.</td></tr>
<tr><td><u>ESMO, 2024</u></td><td>Early-stage</td><td>Diagnosis, Treatment and Follow-Up Guideline

Diagnosis and Imaging (<u>Supplementary Material, Section 3</u>)
MRI recommended in case of uncertainties following standard imaging and in special clinical situations such as:
Familial BC associated w gBRCA1/2m and other high-risk pathogenic variants
Lobular cancers (including detection of extensive multifocality, contralateral BC or to plan conservative surgery)
Suspicion of multifocality and/or multicentricity
Discrepancy b/n conventional imaging and clinical exam
When findings of conventional imaging inconclusive (e.g. positive axillary LN w occult primary tumour)
Presence of breast implants
Staging and Risk Ax (<u>Supplementary Material, Section 3</u>)
Chest, abdomen, and bone imaging recommended for higher-risk pts (high tumour burden, aggressive biology or clinical signs, symptoms or lab values suggesting presence of mets).
F18-FDG–PET–CT scanning may be useful for high-risk pts and when conventional methods inconclusive.
Ax of distant mets (bone, liver, and lung) recommended only in pts w stage IIb and higher disease (esp. w extended LN involvement), pts w high risk of recurrence at first diagnosis and/or in symptomatic pts.
Brain imaging should not be routinely carried out in asymptomatic pts.
Min. imaging work-up for staging includes CT of chest and abdomen and bone scintigraphy. FDG– PET–CT may be used instead of CT and bone scintigraphy.</td></tr>
<tr><td><u>NICE</u>, published in Jul 2018; updated in Jan 2024</td><td>Early and LABC</td><td><u>Preop Assessment Recommendations</u>
Do not routinely use MRI of breast as part of preop Ax of people w biopsy-proven invasive BC or DCIS
Offer MRI of breast to people w invasive BC:
If extent of disease not clear from clinical exam, mammography, and US assessment for planning treatment.
If accurate mammographic assessment difficult b/c of breast density.
To assess tumour size if BCS being considered for ILC.</td></tr>
<tr><td><u>CCO</u>, published in 2019, updated in 2024</td><td>Stage I-III</td><td>Baseline Staging Imaging for Distant Metastasis in Women w Stage I, II, and III BC

<u>Recommendations</u>:
Staging tests using conventional anatomic (chest X-ray, liver US, chest-abdomen-pelvis CT scan) and/or metabolic imaging modalities (PET/CT, PET/MR, bone scintigraphy) should not be ordered routinely for women newly diagnosed w clinical stage I or stage II BC, and w no symptoms of</td></tr>
</table>

**Guideline author, year**	**Stage**	**Staging Imaging for <u>Asymptomatic</u> Pts with Newly Diagnosed, Biopsy Proven BC**
		distant metastasis, regardless of biomarker status.<br>2. **<u>New in 2024</u>:** In women newly diagnosed w stage III BC, baseline staging tests, using PET/CT preferred modality, and should be considered regardless of whether pt is symptomatic for distant mets or not, and regardless of biomarker profile.<br>• If no access to PET, anatomic and/or metabolic imaging modalities may be used.<br>• Based on <u>Dayes et al</u>. RCT, TNM stage III or IIb (T3N0, but not T2N1), n=369 randomized to PET/CT or bone scan and CT CAP<br>•
<u>CCO, 2023</u>		**Clinical Expert Review Re. Role of PET in Clinical Mgmt. of Patients w Cancer**<br><br>**Current PET Indications for BC:**<br>• For staging of pts w histologically confirmed clinical stage 2b or stage 3 BC being considered for curative intent combined modality treatment; and/or repeat PET on completion of neoadj. therapy, prior to surgery (when clinical suspicion of progression)<br><br>Reviewer's Comments: Review not completed by clinical expert in BC.
<u>CCO, 2023</u>	Any stage	**Preop Breast MRI Guideline**<br><br><u>Target Audience</u>: Pts diagnosed w BC of any stage for which additional info on disease location or extent in breast obtained prior to surgery may influence staging, treatment, or prognosis. Guideline does not address pts diagnosed w BC but w/o identified cancerous lesion in breast (occult BC).<br><br><u>Recommendations</u>:<br>1. Preop breast MRI should be considered on case-by-case basis in pts diagnosed w BC for whom additional info re. disease extent could influence treatment. Ensuing decision of whether to conduct MRI should be made in consultation w pt and must consider balance of benefits and risks and pt preferences.<br>2. Preop breast MRI recommended in pts diagnosed w ILC for whom additional info about disease extent could influence treatment. Decision of whether to conduct MRI should be made in consultation w pt and must consider balance of benefits and risks and pt preferences.<br>3. Preop breast MRI recommended, based on opinion of Working Group, in following situations:<br>a) To aid in surgical planning of BCS in pts w suspected or known multicentric or multifocal disease.<br>b) To identify additional lesions in pts w dense breasts.<br>c) To determine presence of pectoralis major muscle/chest wall invasion in pts w posteriorly located tumours or when invasion of pectoralis major muscle or chest wall suspected.<br>d) To aid in surgical planning for skin/nipple-sparing mastectomies or for autologous reconstruction, oncoplastic surgery, and BCS w suspected nipple/areolar involvement.<br>e) Pts w familial/hereditary BC but who have not had recent breast MRI as part of screening or diagnosis.

Guideline author, year	Stage	Staging Imaging for <u>Asymptomatic</u> Pts with Newly Diagnosed, Biopsy Proven BC
<u>American College of Radiology, 2023</u>	Early- and late-stage	**ACR Appropriateness Criteria – Imaging of Invasive BC**<br><br>**Variant 1:** Newly diagnosed. Clinical stage I-IIA (early stage) BC at presentation. Evaluation for locoregional disease (includes invasive ductal carcinoma [IDC], or invasive lobular carcinoma [ILC], or not otherwise specified [NOS]).<br>• <u>Usually appropriate</u> = US breast, digital breast tomosynthesis diagnostic, mammography diagnostic, MRI breast w/o and w IV contrast.<br>• <u>May be appropriate</u> = US axilla, mammography w IV contrast.<br>• <u>Usually not appropriate</u> = MRI breast w IV contrast, bone scan whole body, CT CAP w IV contrast, CT CAP w/o and w IV contrast, CT CAP w/o IV contrast, FDG-PET/CT skull base to mid-thigh.<br><br>**Variant 2:** Newly diagnosed. Clinical stage I-IIA (early stage) BC at presentation. Evaluation for distant disease (includes IDC, or ILC, or NOS).<br>• <u>Usually not appropriate</u> = US axilla, US breast, digital breast tomosynthesis diagnostic, mammography diagnostic, mammography w IV contrast, MRI breast w/o and w IV contrast, MRI breast w/o IV contrast, bone scan whole body, CT CAP w IV contrast, CT CAP w/o and w IV contrast, CT cheat abdomen pelvis w/o IV contrast, FDG-PET/CT skull base to mid-thigh.<br><br>**Variant 3:** Newly diagnosed. Clinical stage IIB-III (late stage) BC at presentation. Evaluation for locoregional disease (includes IDC, or ILC, or NOS).<br>• <u>Usually appropriate</u> = US axilla, US breast, digital breast tomosynthesis diagnostic, mammography diagnostic, MRI breast w/o and w IV contrast, FDG-PET/CT skull base to mid-thigh.<br>• <u>May be appropriate</u> = mammography w IV contrast.<br>• <u>Usually not appropriate</u> = MRI breast w/o IV contrast, bone scan whole body, CT CAP w IV contrast, CT CAP w/o and w IV contrast, CT CAP w/o IV contrast.<br><br>**Variant 4:** Newly diagnosed. Clinical stage IIB-III (late stage) BC at presentation. Evaluation for distant disease. IDC or ILC that is ER+/HER2-.<br>• <u>Usually appropriate</u> = bone scan whole body, CT CAP w IV contrast, FDG-PET/CT skull base to mid-thigh.<br>• <u>Usually not appropriate</u> = US axilla, US breast, digital breast tomosynthesis diagnostic, mammography diagnostic, mammography w IV contrast, MRI breast w/o and w IV contrast, CT CAP w/o and w IV contrast, CT CAP w/o IV contrast.<br><br>**Variant 5:** Newly diagnosed. Clinical stage IIB-III (late stage) BC at presentation. Evaluation for distant disease. IDC or ILC that is HER2+ or triple negative (ER, PR, and HER2-).<br>• <u>Usually appropriate</u> = bone scan whole body, CT CAP w IV contrast, FDG-PET/CT skull base to mid-thigh.<br>• <u>Usually not appropriate</u> = US axilla, US breast, digital breast tomosynthesis diagnostic, mammography diagnostic, mammography w IV contrast, MRI breast w/o and w IV contrast, MRI breast w/o IV contrast, MRI head w/o and w IV contrast, MRI head w/o IV contrast, CT CAP w/o and w IV contrast, CT CAP w/o IV contrast.

Guideline author, year	Stage	Staging Imaging for Asymptomatic Pts with Newly Diagnosed, Biopsy Proven BC
SEOM (Spanish Group), 2023	Early-stage	**SEOM-GEICAM-SOLTI clinical guidelines for early-stage BC (2022)**<br><br>***Diagnosis and staging***<br>• Breast MRI optional (I, B) and should be only considered in cases of positive axillary nodes; occult primary BC; Paget's disease of nipple; lobular carcinoma; multifocal, multicentric lesions, and BC implants. Also recommended pre- and post-neoadj. treatment to define extent of disease and monitor treatment response (III, A).<br>• Additional systemic staging should be contemplated when disease detected in stage III or when signs, symptoms, or laboratory values indicate possible metastasis. This more comprehensive study includes chest, abdominal, and pelvic imaging, and bone scan (III, B). PET/CT may be of use when traditional imaging test are inconclusive (III, A) or in cases of locally advanced tumours.
ESMO, 2021	Metastatic	Staging Recommendations**:**<br>1. Min. imaging work-up for staging includes CT of chest and abdomen and bone scintigraphy [II, A].<br>2. ^{18}F-FDG PET-CT may be used instead of CT and bone scans [II, B].<br>3. No evidence that any staging or monitoring approach provides OS benefit over another.<br>4. Impending fracture risk should be evaluated by CT or X-rays. Spine instability neoplastic score provides reproducible risk assessment for vertebral mets. In case of suspected cord compression MRI modality of choice [I, A].<br>5. Brain imaging should not be routinely carried out in all asymptomatic pts at initial MBC diagnosis. Pts w asymptomatic HER2+ BC or TNBC have higher rates of brain mets at initial diagnosis, even as first site of recurrence. This may warrant subtype-oriented brain imaging in asymptomatic pts w MBC if detection of CNS mets will alter choice of systemic therapy [V, C].
German Guidelines, 2021	All stages	• In case of newly diagnosed BC from UICC stage II w increased risk and III and IV w/o symptoms of metastasis, staging (lung, liver, skeleton) should be performed [2a, B]<br>• In case of newly diagnosed BC and clinical suspicion of mets, imaging staging shall be performed [2a, A]<br>• Whole body staging should only be performed in women w higher risk of metastasis (N+, >T2) and/or aggressive tumour biology (e.g., HER2+, TN), clinical, signs, symptoms and if planned decision on systemic chemo/antibody therapy planned. Whole body staging should be performed using CT thorax/abdomen and skeletal scintigraphy. [EC]
ESMO, 2020	Advanced BC	ABC image guideline statements:<br>• Imaging of chest, abdomen, and bones. [II/A]<br>• Brain imaging should not be routinely performed in asymptomatic pts. Approach applicable to all pts w ABC, including those w HER2+ and/or advanced TNBC. [II/D]<br><br>LABC (inoperable, non-metastatic LABC) guidelines statements:<br>• Imaging of chest and abdomen (preferably CT scan) and bone before initiation of systemic therapy highly recommended [I/A]<br>• PET-CT, if available, may be used for LABC pts (instead of and not in addition to CT scans and bone scan) [II/B]

**Guideline author, year**	**Stage**	**Staging Imaging for Asymptomatic Pts with Newly Diagnosed, Biopsy Proven BC**
American College of Radiology, 2019	Stage I	**From abstract:** National and international guidelines discourage use of staging imaging for asymptomatic pts newly diagnosed w stage 0 to II BC, even if nodal involvement, as unnecessary imaging can delay care and affect outcomes. In asymptomatic pts w history of stage I BC that received Tx for curative intent, no role for imaging to screen for distant recurrences.<br><br>**Summary of recommendations for initial imaging of newly diagnosed asymptomatic women w stage I BC**<br>• Imaging not recommended to rule out bone mets.<br>• Imaging not recommended to rule out thoracic mets.<br>• Imaging not recommended to rule out abdominal mets.<br>• Imaging not recommended to rule out brain mets.
ESMO, 2019	Early BC	Staging Recommendations:<br>• Routine staging evaluations directed at locoregional disease. Asymptomatic distant mets rare, and most pts do not benefit from comprehensive lab tests (including tumour markers) and radiological staging [III, D].<br>• CT scan of chest, abdominal imaging (US, CT, or MRI scan) and bone scan can be considered for pts w:<br>o Clinically positive axillary nodes<br>o Large tumours (e.g., ≥5 cm)<br>o Aggressive biology<br>o Clinical signs, symptoms or lab values suggesting presence of mets [III, A]<br>• Dual imaging methods combining functional and anatomical info such as FDG PET-CT may be useful when conventional methods inconclusive [V, A].<br>• PET-CT scanning can also replace traditional imaging for staging in high-risk pts [V, B], although in cases of lobular cancers and low-grade tumours, PET-CT may be less sensitive.<br>• Use of FDG-PET-CT not supported in staging of locoregional disease, due to its limited sensitivity when compared w gold standard, SLNB and ALND.

Supplementary Table S2. Summary of white literature used to draft premeeting survey (published between 2013 – Nov 6, 2023)

Author, date (Level of evidence)	n	Imaging Modality	Pt Inclusion	Pt Characteristics	Confirmation of Lesions on Imaging	Unsuspected Distant Mets	Upstaged to IV	Change in Mgmt.
Dayes, 2023 (RCT - I)	369	PET-CT n=184	Histological evidence of invasive DCIS and stage III by TNM (T0N2, T1N2, T2N2, T3N1,2 or T4) or IIb BC (T3N0) on basis of clinical info and being considered for combined modality therapy of curative intent.	Stage<br>- IIB/IIIA/IIIB/IIIC, %: 28/50/20/2<br>- ER+/PR+/Her2Neu+, %: 70/52/35	Histology or imaging	23.4%<br><br>Most common:<br>- Bone only, n=14<br>- Bone, liver, n=6	23% of PET-CT pts upstaged to stage IV<br><br>vs.<br><br>11% conventional staged pts (RR, 2.4 [95% CI, 1.4 to 4.2]; p=0.002	TX changed in 81.3% of upstaged PET-CT pts and 95.2% of upstaged conventional pts.
		BS, CT C/A/P n=185		Stage<br>- IIB/IIIA/IIIB/IIIC, %:23/56/19/2<br>- ER+/PR+/Her2Neu+, %: 72/54/32		11.4%<br><br>Most common:<br>- Bone only, n=6<br>- Bone, lung, n=5		
Bruckmann, 2021 (Prospective – III)	80	Thoraco-abdominal ceCT and whole-body 18F-FDG PET/ MRI	1) newly diagnosed, treatment-naive T2-tumor or higher T-stage <u>or</u><br><br>2) newly diagnosed, treatment-naive triple-negative tumor of every size <u>or</u><br><br>3) newly diagnosed, treatment-naive tumor w molecular high risk (T1c, Ki67 >14%, HER2neu over-expression, G3)	NR	Histology and imaging	Distant mets present in 8.75% (n=7/80)<br><br>18 F-FDG PET/MRI detected all histopathological proven mets w/o any false-positive findings, while 3 pts w bone mets missed in CT (sensitivity 57%, specificity 96%)<br><br>Additionally, CT presented false-positive findings in 3 pts.	NR	NR

**Author, date (Level of evidence)**	**n**	**Imaging Modality**	**Pt Inclusion**	**Pt Characteristics**	**Confirmation of Lesions on Imaging**	**Unsuspected Distant Mets**	**Upstaged to IV**	**Change in Mgmt.**
Bruckman, 2021 (Prospective – III)	154	Whole-body [18F]FDG PET/MRI, CT scan, and BS prior to therapy	Therapy-naive pts w newly diagnosed, histopathologically proven BC	- PR+, 70%<br>- ER+, 75%<br>- Ki 67+ (>14%), 92%<br>- HER2-neu 0/1+/2+, 3+, %: 36/32/15/17<br>- Luminal A/B/HER2-enriched/Basal-like, %: 5/75/2/18<br>- IDC/ILC/Mixed, %: 88/8/1	Histology	41 bone mets present in 4.5% pts (n=7)<br><br>Both [18F]FDG PET/MRI and MRI alone able to detect all pts w bone mets (sensitivity 100%; specificity 100%) and did not miss any of the 41 malignant lesions (sensitivity 100%)<br><br>CT detected 5/7 pts (sensitivity 71.4%; specificity 98.6%) and 23/41 lesions (sensitivity 56.1%)<br><br>BS detected only 2/7 pts (sensitivity 28.6%) and 15/41 lesions (sensitivity 36.6%).<br><br>CT and BS led to false-positive findings of bone mets in 2 pts and in 1 pt, respectively	NR	NR

Author, date (Level of evidence)	n	Imaging Modality	Pt Inclusion	Pt Characteristics	Confirmation of Lesions on Imaging	Unsuspected Distant Mets	Upstaged to IV	Change in Mgmt.
						Sensitivity of PET/MRI and MRI alone significantly better compared w CT ($p < 0.01$, difference 43.9%) and bone scintigraphy ($p<0.01$, difference 63.4%)		
Bruckman, 2020 (Prospective – III)	104	Whole-body ^{18}F-FDG PET/MRI	Newly diagnosed, histopathologically proven BC	- PR+, 71%<br>- ER+, 74%<br>- Ki 67+ (>14%), 85%<br>- HER2-neu 0/1+/2+, 3+, %: 40/32/11/17<br>- Luminal A/B/HER2-enriched/Basal-like, %: 12/71/2/15<br>- IDC/ILC/Mixed, %: 93/5/1	Histology (majority)	M stage identified correctly in MRI and ^{18}F-FDG PET/MRI in 96% (n=100)<br>Both modalities correctly staged all 7 pts w distant mets, leading to false-positive findings in 4 pts in each modality (3.8%)<br><br>In lesion-based analysis, ^{18}F-FDG PET/MRI showed significantly better performance in correctly determining malignant lesions (85.8% vs. 67.1%, difference 18.7% (95% CI: 0.13-0.26), $p<0.0001$) and offered superior diagnostic confidence compared w MRI alone (4.1 ± 0.7 vs. 3.4 ± 0.7, $p<0.0001$)	NR	NR

Author, date (Level of evidence)	n	Imaging Modality	Pt Inclusion	Pt Characteristics	Confirmation of Lesions on Imaging	Unsuspected Distant Mets	Upstaged to IV	Change in Mgmt.
Han, 2021 (Meta-analysis nonrandomized – III)	4276	18F-FDG PET<br><br>PET/CT<br><br>PET/MRI	Newly diagnosed BC	Not summarized	Histology or imaging	NR	For all 24 studies (26 cohorts) combined, pooled proportion of changes in stage 25% (95% CI, 21%–30%).<br><br>Substantial heterogeneity based on Higgins I^2 statistics (I^2=89%).	For all 22 studies (24 cohorts), pooled proportion 18% (95% CI, 14%–23%).<br><br>Substantial heterogeneity based on Higgins I^2 statistics (I^2=87%)
Jeong, 2024 (Retro – IV)	2056	Abdominal ceCT	Newly diagnosed pathologically-confirmed BC	Pathology<br>- IDC/ILC/Others, %: 92/4/4<br><br>Stage<br>- 0/I/II/III, %: 1/20/56/23<br><br>LN+/ER+/PR+/HER2+, %: 29/68/53/27<br><br>Subtype<br>Luminal/HER2-enriched/TNBC, %: 53/27/20	Histology or imaging	Total diagnostic yield, false-referral rate, and PPV for BC liver metastasis 1.1%, 0.1%, and 91.7%<br><br>Diagnostic yield higher in stage III cancers than in stage I or II cancers (3.9% vs. 0% or 0.4%, p<0.001), and in HER-2 enriched cancers than in luminal or triple-negative cancers (2.9% vs. 0.4% or 0.5%, p<0.001)	5.2%	NR

Author, date (Level of evidence)	n	Imaging Modality	Pt Inclusion	Pt Characteristics	Confirmation of Lesions on Imaging	Unsuspected Distant Mets	Upstaged to IV	Change in Mgmt.
						26 had metastasis other than BC liver metastasis on abdominal CT: 20 bony systems, 5 multiple organs, and 1 LN		
Leem, 2023 (Retro - IV)	153	CT C/A/P	Stage II and III<br><br>Focus on stage II ≥N1	Stage II (n=102)<br>- T1/2/3/4, %: 12/51/37/0<br>- ER+/PR+/HER2+, %: 69/60/20<br>- ≥N1, %: 86<br><br>Stage III (n=51)<br>- T1/2/3/4, %: 14/37/49/14<br>- ER+/PR+/HER2+, %: 71/55/16	Distant metastasis confirmed as either suspicious nodules w radiological evidence of progression or by histology	Stage II, 7.8%<br>- 6/8, cN1<br>- 2/8, cN0<br><br>Stage III, 18%	6.8% stage II node+ pts<br><br>17% stage III	NR
Glecker, 2023 (Retro - IV)	290	MRI	All newly diagnosed operable BC	Pathology<br>- DCIS/IDC/ILC/Mixed IDC/ILC, %: 26/60/11/2<br><br>Stage<br>- 0/IA/IB/IIA/IIB/IIIA/IIIB/IIIC/IV, %: 1/29/45/11/10/1/2/1<br><br>LN+/ER+/PR+/HER2+, %: 10/88/79/7	Histology or imaging	18% extramammary findings (most frequent sites liver 64% and kidney 13%)<br><br>1 malignancy identified in thymus. No metastatic BC.<br><br>Regional lymphadenopathy, such as axillary, internal mammary, or supraclavicular LNs, not considered extramammary findings	0	NR

Author, date (Level of evidence)	n	Imaging Modality	Pt Inclusion	Pt Characteristics	Confirmation of Lesions on Imaging	Unsuspected Distant Mets	Upstaged to IV	Change in Mgmt.
Kamel, 2021<br>(Retro - IV)	295	CT n=152,<br><br>BS=156<br><br>Abdo US n=12<br><br>CXR n=12	T1-T2, N0/N1<br><br>168/295 pts had total of 332 baseline radiological staging tests	T stage<br>- Ta1/1c/T2, %: 17/31/52<br><br>N1: 27%<br><br>TNM stage<br>- I/IIA/IIB, %: 42/37/21<br><br>ER and/or PR+/HER2+/TNBC, %: 84/13/12	Histology or imaging	Overt metastatic disease:<br>CT: 4/152<br>BS: 1/156<br><br>Nonspecific findings requiring follow-up:<br>CT: 68/152<br>BS: 19/156<br>Abdo US: 2/12<br><br>Triggered 138 follow-up scans; 9 pts underwent biopsy of suspicious lesions:<br>- 1 metastatic BC<br>- 2 early-stage lung cancer<br>- 1 gastric schwannoma after multiple investigations	NR	NR
Nam, 2021<br>(Retro - IV)	1002	A/P CT	EBC, n=673<br>ABC n=268<br>RBC n=43 included if A/P CT performed in absence of clinical evidence of metastasis in abdominopelvic area	Entire cohort<br>- IDC/ILC/IDC+ILC, %: 79/17/2<br>- Stage I/II/III/IV, %: 23/44/22/1<br>- NACT 3.8%<br>- ER+/PR+/HER2+/LN+, %: 53/54/27/25<br><br>EBC Stage I/II, %: 34/66<br><br>ABC: Stage III/IV, %: 81/5	Imaging	EBC 3.1%<br>ABC 13.8%<br>RBC 16.3%<br><br>69 metastatic lesions in 65 pts:<br>- bone, n=33<br>- LN, n=20<br>- Liver, n=13<br>- Other sites, n=3	2.9% pts upstaged to stage IV:<br>- 4 from stage I<br>- 17 from stage II<br>- 8 from stage III	

Author, date (Level of evidence)	n	Imaging Modality	Pt Inclusion	Pt Characteristics	Confirmation of Lesions on Imaging	Unsuspected Distant Mets	Upstaged to IV	Change in Mgmt.
				NACT (14%)		*For prevalence of mets by site and mets by stage (upstaged to IV), numbers based on entire cohort, which also included 43 recurrent BC pts; no way to tease out		
Hyland, 2020 (Retro – IV)	799	PET/CT (n=298) or<br><br>C/A/P CT and full-body bone scan (standard of care imaging (n=231)	Clinical stage II–III invasive BC, tumours ≥2.5 cm, and high-risk molecular features (I-SPY2 pts)	Standard of Care<br>- IDC/ILC, %: 90/7<br>- HR+/HER2-, 48%<br>- HR+ or HR-/HER2+, 18%<br>- HR-/HER2-, 32%<br>- LN+, 42%<br><br>PET/CT<br>- IDC/ILC, %: 93/5<br>- HR+/HER2-, 51%<br>- HR+ or HR-/HER2+, 25%<br>- HR-/HER2-, 24%<br>- LN+, 39%	NR	De novo metastatic disease rate 4.6% (37/799)<br><br>False positive rate higher using standard of care vs PET/ CT (22.1% vs 11.1%; P=0.0009)<br><br>Mean time b/n incidental finding on baseline imaging to FP determination 10.8 days<br><br>Mean time from diagnosis to chemo initiation 44.3 days w standard of care vs. 37.5 days w PET/CT (P=0.0001)	NR	NR
Rajasooriyar, 2020 (Retro – IV)	233	C/A/P CT	Asymptomatic BC w normal CXR and US of abdomen but later upstaged to stage IV by C/A/P CT	Tx/1/2/3/4, %: 1/21/48/17/13<br>N0/1/2/3/, %: 42/27/16/15<br>Ductal/lobular, %: 94/3<br>ER+/PR+/HER2+, %: 38/34/27	Imaging	34 pts (15%) had occult metastasis on CT. 44 abnormalities detected.<br><br>Pts w occult metastasis:	T1=4%<br>T2/3/4=17%<br><br>N0=5%<br>N1-3=21%	NR

Author, date (Level of evidence)	n	Imaging Modality	Pt Inclusion	Pt Characteristics	Confirmation of Lesions on Imaging	Unsuspected Distant Mets	Upstaged to IV	Change in Mgmt.
						- 0 stage I<br>- 11 stage II<br>- 24 stage III<br><br>Of these pts:<br>- 3/56 (5%) stage IIA<br>- 8/48 (17%) of stage IIB<br>- 23% (n = 23/100) of stage III pts had occult metastasis on CT scan<br><br>Most common sites of metastasis: bone (n=25) lung (n=10), liver (n 6), and others (n=3).	I/IIA = 3.8<br>IIB/III=20%	
James, 2019<br><br>(Retro – IV)	335	Chest CT	EBC (I/II), n=499<br>ABC (III), n=98<br>RBC, n=56	DCIS/IDC/ILC, %: 8/77/12/3<br>Stage 0/I/II/III/IV, %: 7/31/38/8/1<br>NACT, 4%<br>Grade I/2/3, %: 14/36/36/15<br>ER+/PR+/HER2+/Node+, %: 85/74/13/39	Histological	4% new metastases, and 1 incidental lung cancer<br><br>85% of all scans only showed benign or normal findings, while 10% had indeterminate findings identified as false positive after further tests or observation<br><br>EBC	NR	NR

Author, date (Level of evidence)	n	Imaging Modality	Pt Inclusion	Pt Characteristics	Confirmation of Lesions on Imaging	Unsuspected Distant Mets	Upstaged to IV	Change in Mgmt.
						- 200 scanned, 2% true positive (3 lung, 1 bone/LN)<br><br>ABC<br>- Stage III, 95% scanned, 3% true positive<br>- Stage IV, 100% scanned, 86% true positive<br>- NACT, 95% scanned, 8% true positive		
Srour, 2020 (Retro – IV)	262	PET/CT, n=217<br><br>C/A/P CT, n=45	Stage I-III invasive BC who had NAC w subsequent breast operation	- IDC/ILC/Mixed invasive, %: 92/6/2<br>- ER+/HER2+/TNBC, %: 31/42/25<br>- T0/1/2/3/4, %: 1/19/55/15/11<br>- N0/1/2/3, %: 39/58/1/3		55.7% pts had 222 incidental radiologic findings on PET/CT scan:<br>- 1 finding by Stage 1/2/3, %: 30/39/25<br>- 2 findings by Stage 1/2/3, %: 19/16/16<br>- ≥3 findings by Stage 1/2/3, %: 7/5/7<br><br>Most incidental radiologic findings:<br>- Ovary/uterus 30%<br>- Lung 18%<br>- Liver 10%<br>- Bone 9%<br><br>At median follow-up 3.7 yrs., 15.6% pts had distant recurrence.	NA	NA

Author, date (Level of evidence)	n	Imaging Modality	Pt Inclusion	Pt Characteristics	Confirmation of Lesions on Imaging	Unsuspected Distant Mets	Upstaged to IV	Change in Mgmt.
						Most common sites of distant mets: bone 37%, lung 19%, liver 16%, and brain 16%, and multiple concurrent organ systems 7%.<br><br>Of these pts, only 1.9% pts had distant metastasis in same organ initially thought to be incidental radiologic finding in bone (n=3) and lung (n=2)<br><br>Of these, 5 pts had stage 2 (n=2, 40%) and stage 3 (n 3, 60%) disease		
Srour, 2019 (Retro – IV)	303	PET/CT, n=258<br><br>Brain, n=94<br><br>BS, n=117<br><br>All above, n=48	Stage 1 or 2 BC who had NACT w subsequent breast operation	IDC/ILC/Mixed, %: 92/5/2<br>ER+/HER2+/TNBC, %: 30/43/27<br>T0/1/2/3/4, %: 1/22/71/6/0<br>N0/1, %: 49/51<br><br>20.7% of pts who underwent staging imaging were symptomatic.	Imaging	NR	4.9% w positive PET/CT upstaged to Stage IV; only 2/15 symptomatic before staging imaging:<br>- T1=7%<br>- T2=9%<br>- T3=7%<br>- N0=7%	NR

Author, date (Level of evidence)	n	Imaging Modality	Pt Inclusion	Pt Characteristics	Confirmation of Lesions on Imaging	Unsuspected Distant Mets	Upstaged to IV	Change in Mgmt.
							- N1=93%	
Bansal, 2018<br>(Retro – IV)	105	BS<br>C/A/P CT	LABC (N2, N3, T4) and locally recurrent BC pts. Other inclusion criteria included both imaging investigations performed either on same day or w/n 10 days of each other	NR	Clinico-radiological and histological follow-up	31.4% had concordant normal results on CT and BS<br><br>17.1% pts w extraosseous metastasis on CT w negative or inconclusive BS<br><br>Bone scans diagnosed peripheral osseous metastasis in 4.7%, which were either skull or extremity metastasis outside CT TAP field of view.	NR	4.7% of pts w peripheral osseous metastasis had other metastatic lesions w/n axial skeleton or soft tissues on CT and led to no change in pt mgmt.
Shah, 2018<br>(Retro – IV)	150	Breast MRI	All pts who underwent breast MRI (n=7070), then all pts in whom hepatic lesion described in impression of report for which further imaging recommended (n=201)	84.7% pts had T2 hyperintense liver lesion<br><br>Newly diagnosed BC, n=39<br>High risk screening, n=35<br>Prior history of BC, n=56<br>Misc, n=20	Dedicated abdo imaging (US, CT, MRI or PET/CT)<br><br>Histology	N=4 (2.7%) T2 hyperintense lesions characterized as malignant, and specifically, liver mets from BC, at dedicated abdominal imaging<br><br>Indication for imaging in all 4 cases was newly diagnosed BC	NR	NR

Author, date (Level of evidence)	n	Imaging Modality	Pt Inclusion	Pt Characteristics	Confirmation of Lesions on Imaging	Unsuspected Distant Mets	Upstaged to IV	Change in Mgmt.
			51 pts excluded b/c of lack of further imaging/ follow-up			Of these, 3 had biopsy-proven metastatic disease. The fourth did not have a biopsy at study institution.		
Dull, 2017 (Retro – IV)	3,321	Chest CT (n=683)<br><br>(Stage 1, 11%/Stage 2 36%)	Stage I (62%) and II (38%) no NACT	In pts w staging chest CT (n=683)<br><br>Stage !/II, %: 33/67<br>T1/2/3, %: 52/45/3<br>N0 (incl. N1mic)/N1, %: 64/35<br>ER+/PR+/HER2+, %: 67/60/22	Routine follow-up chest CTs	1.3% pulmonary mets at avg. of 25 mos. after initial staging chest CT; n= 2 Stage 1 and n=7 stage II	0.4% at initial staging CT	NR
James, 2017 (Retro – IV)	586	C/A/P CT (n=285, 49%)<br><br>Indicated for: (1) node pos. (2) selected for NAC (3) high risk node neg. EBC selected for adj. cytotoxic/ targeted systemic therapy	Pts w primary invasive BC who underwent BC treatment	49%<br><br>IDC/ILC/Mixed, %: 76/13/2<br><br>IA/IB/IIA/IIB/IIIA/IIIC/NACT, %: 37/2/28/20/7/3/5<br><br>ER+/PR+/HER2+, %: 84/74/13/	NR	1% (n=4) asymptomatic synchronous metastasis detected	NR	NR

Author, date (Level of evidence)	n	Imaging Modality	Pt Inclusion	Pt Characteristics	Confirmation of Lesions on Imaging	Unsuspected Distant Mets	Upstaged to IV	Change in Mgmt.
Lebon, 2017 (Retro – IV)	214	18F-FDG PET/CT	<40 yrs., n=107<br>≥40 yrs., n=107	<40 yrs.<br>- Stage I/IIA/IIB/III, %: 11/30/28/31<br>- HR+/HER2-, 34%<br>- HER2+, 33%<br>- TNBC, 33%<br><br>≥40 yrs.<br>- Stage I/IIA/IIB/III, %: 11/30/28/31<br>- HR+/HER2-, 50%<br>- HER2+, 24%<br>- TNBC, 26%	Interpreter who was unaware of original PET/CT report or any other imaging, follow-up imaging, and pathology for small number of pts	<40 yrs. = 21% (23/107)<br>- bone (n=11), lung (n=2), distant LN (n=6)<br><br>≥40 yrs. = 22% (24/107)<br>- bone (n=7), liver (n=3), lung (n=1), distant LN (n=6)	<40 yrs.<br>- Stage I, 8%<br>- Stage IIA, 9%<br>- Stage IIB, 17%<br>- Stage III, 42%<br><br>≥40 yrs.<br>- Stage I, 8%<br>- Stage IIA, 13%<br>- Stage IIB, 13%<br>- Stage III, 45%	NR
Aliyev, 2016 (Retro – IV)	254	FDG-PET/CT	Group 1: diagnosed by tru-cut/core/FNAB, n=154<br><br>Group 2: diagnosed by excisional biopsy n=32<br><br>Group 3: mastectomy-ALND, n=62<br><br>Group 4: axillary lymph node metastasis diagnosed by excisional biopsy, n=6	- T1/2/3/4, %: 26/51/8/15<br>- IDC/ILC/mixed/mucinous, %: 80/5/6/2	Histopathological exam, radiological correlation, and clinical and PET imaging follow-ups.	29.9% (n=76)<br><br>Of these:<br>- 22% bone/bone marrow<br>- 7% lung<br>- 13% mediastinal LN<br>- 5% liver<br>- 10% had other organ/system<br>- 6% contralateral axillary–supraclavicular–internal mammary lymphadenopathies<br><br>According to T stage, rate of distant mets:<br>- 14% T1<br>- 36% T2	NR	NR

Author, date (Level of evidence)	n	Imaging Modality	Pt Inclusion	Pt Characteristics	Confirmation of Lesions on Imaging	Unsuspected Distant Mets	Upstaged to IV	Change in Mgmt.
						- 40% T3<br>- 33% T4 stage		
Bychkovsky, 2016<br>(Retro – IV)	411	Body CT (torso, chest or abdo), n=237	Stage II BC	Stage IIA/IIB, %: 63/37<br>Ductal/lobular/both, %: 68/11/16<br>ER/PR+, 66%<br>HER2+, 17%<br>TNBC, 17%	Histology	Rate of detection of true metastatic disease 2.1% (95% CI, 0.7%–5%); 4 liver, 1 lung<br><br>- 2.2% for ER/PR+<br>- 1.9% for HER21<br>- 2.1% for TNBC	NR	NR
Ulaner, 2016<br>(Retro – IV)	483	18F–FDG-PET/CT	Stage I to IIIC ER+/HER2-, n=238<br><br>Stage I to IIIC HER2+ prior to beginning treatment w chemo, hormonal therapy, or RT, n=245	ER+/HER2-<br>- Stage I/IIA/IIB/IIIA/IIIB/IIIC, %: 6/30/40/10/11/8<br>- IDC/ILC/Mixed, %: 79/14/6<br>- PR+/ER+, %: 86/100<br><br>HER2+<br>- Stage I/IIA/IIB/IIIA/IIIB/IIIC, %: 9/29/38/13/9/2<br>- IDC/ILC/Mixed, %: 92/2/4<br>- PR+/ER+, %: 53/67	Histology	ER+/HER2 unsuspected distant mets in:<br>- 4% initial stage IIA<br>- 14% stage IIB<br>- 26% stage III<br><br>HER2+ unsuspected distant metastases in:<br>- 4% initial stage IIA<br>- 14% stage IIB<br>- 22% stage III	14% pts w both ER+/HER2- and HER2+ initial clinical stage IIB upstaged to stage IV	1 stage IIB HER2+
Ulaner, 2016<br>(Retro – IV)	232	18-F-FDG/PET/CT	TNBC	I/IIA/IIB/IIIA/IIIB/IIIC, %: 10/35/38/10/6/1<br><br>IDC/ILC/Mixed, %: 94/1/2	Histology (n=26), follow-up imaging (n=4)	Unsuspected distant mets in 13%:<br>- 0/23 initial stage I<br>- 4/82 stage IIA<br>- 13/87 stage IIB<br>- 4/23 stage IIIA<br>- 8/14 stage IIIB<br>- 1/3 (33%) stage IIIC.<br><br>Bone, n=11	11% pts upstaged to Stage IV<br><br>7 unsuspected synchronous malignancies identified in 6 pts	NR

Author, date (Level of evidence)	n	Imaging Modality	Pt Inclusion	Pt Characteristics	Confirmation of Lesions on Imaging	Unsuspected Distant Mets	Upstaged to IV	Change in Mgmt.
						Liver, n=8<br>Distant nodes, n=8<br>Lung, n=7<br>Pleura, n=1	Initial stage 2B pts upstaged to 4 by significantly shorter survival compared to initial stage 2B pts	
Hogan, 2015 (Retro – IV)	146	18-F-FDG/PET/CT	ILC, n=146	ILC<br>- Stage I/II/III, %: 5/35/60<br>- ER+/HER2-, 90%<br>- HER2+, 6%<br>- TNBC, 3%<br><br>ILC<br>- Stage I/II/III, %: 0/0100<br>- ER+/HER2-, 52%<br>- HER2+, 21%<br>- TNBC, 26%	Histology	8% of ILC cohort:<br>- 0 w stage I<br>- 4% w stage II<br>- 11% w stage III	3/12 upstaged pts upstaged only by CT component of PET/CT, as mets not 18F-FDG–avid<br><br>In comparison, in stage III IDC cohort, 22% of pts upstaged to IV by PET/CT	NR
Linkugel, 2015 (Retro – IV)	3291	CT, BS PET (n=882)	Stage I, n=2044; 312 imaged<br><br>Stage II, n=1247; 570 imaged	In pts w staging studies performed:<br>- Stage IA/IB/IIA/IIB, %: 33/2/37/28<br>- T1/T2/T3, %: 54/43/4<br>- N0/N1, %: 58/41<br>- ER+/PR+/HER2+, %: 23/55/23	Imaging or histology	194/882 pts, (22%) required additional imaging and/or biopsies to further evaluate abnormalities	NR	NR

**Author, date (Level of evidence)**	**n**	**Imaging Modality**	**Pt Inclusion**	**Pt Characteristics**	**Confirmation of Lesions on Imaging**	**Unsuspected Distant Mets**	**Upstaged to IV**	**Change in Mgmt.**
						Only 11 (5%) confirmed to have metastasis (1.2% of imaged pts, 0.3% of total cohort). Of these, 1 was stage I at presentation and 10 were stage II<br><br>Sites of distant metastasis: lung (n=3), bone (n=4), liver (n=1), and combination of previously mentioned sites (n=3)		
Louie, 2015 (Retro – IV)	1043	CXR	Stage I/II, n=959<br>Stage III, n=84	Stage I/II/III, %: 59/30/11	Imaging	Stage I/II 0%<br>Stage III 2.4%	NR	NR
Chen, 2014 (Retro – IV)	3411	Bone Scan, liver US, chest x-ray	Stage I-III	- Stage I/II/III, %: 12/75/13<br>- cT2/T2/T3/T4, %: 39/55/6/1<br>- cN0/1/2/3, %: 47/31/14/9<br>- ER+/PR+/HER2+/Ki67 (>14%), %: 61/67/23/61	Bone mets indicated by BS confirmed by CT or MRI; liver mets indicated by LUS confirmed by liver dual phase scan CT; lung mets indicated by chest X-ray confirmed by chest CT or MRI	Number of pts w mets not reported<br><br>Bone, n=46<br>- Stage I/II/II: 5/33/8<br><br>Liver, n=14<br>- Stage I/II/II: 2/10/2<br><br>Lung, n=7<br>- Stage I/II/II: 1/5/1	Stage I, 2%<br>Stage II, 2%<br>Stage III, 3%	NR

Author, date (Level of evidence)	n	Imaging Modality	Pt Inclusion	Pt Characteristics	Confirmation of Lesions on Imaging	Unsuspected Distant Mets	Upstaged to IV	Change in Mgmt.
Cochet, 2014 (Prospective – III)	142	^{18}F-FDG PET/CT	Stage IIA-IV	- Stage IIA/IIB/IIIA/IIIB/IIIC/IV, %: 15/40/9/13/11/12<br>- ER+/PR+/HER2+, %: 63/56/44<br>- IDC/ILC, %: 90/8<br>- Luminal A: 36%<br>- Luminal B/HER2-, 8%<br>- Luminal B/HER2+, 23%<br>- HER2+ (nonluminal) 11%<br>- TNBC, 22%	Imaging and clinical follow-up and/or pathology	18%<br>- bone, n=15<br>- liver, n=4<br>- lung, n=3<br>- distant LN, n=4	Stage IIA, 9%<br>Stage IIB, 7%<br><br>Stage IIIA, 0%<br>Stage IIIB, 21%<br>Stage IIIC, 13%	11 (8%) from curative to palliative; 4(3%) from palliative to curative treatment after PET/CT suggested absence of distant mets
Debalt, 2014 (Retro – IV)	742	Chest X-ray, liver US, bone scan	Newly diagnosed BC w full initial staging and no symptoms of mets	- pT1/T2/T3/T4/Tx, %: 63/28/4/4/1<br>- pN0/1/2/3/4/Nx, %: 56/21/10/5/7<br>- G1/2/3/Gx, %: 6/59/34/1<br>- IDC/ILC/Combined/Others, %: 67/17/9/8<br>- ER+/PR+/HER2+, %: 78/73/14	Imaging	1.2% distant mets; 38.8% suspicious vs. 2% confirmed distant mets w additional imaging<br><br>Chest X-ray<br>- 80% sensitivity,<br>- 93.5% specificity<br>- 7.7% PPV<br><br>Liver U/S<br>- 100% sensitivity<br>- 92.7% specificity<br>- 5.3% PPV<br><br>Bone scan<br>- 100% sensitivity<br>- 70.1% specificity<br>- 5.6% PPV	NR	NR

Author, date (Level of evidence)	n	Imaging Modality	Pt Inclusion	Pt Characteristics	Confirmation of Lesions on Imaging	Unsuspected Distant Mets	Upstaged to IV	Change in Mgmt.
Jeong, 2014 (Retro – IV)	178	F-18 FDG PET/CT	Clinical negative axillary nodal involvement	- IDC/ILC/DCIS, %:82/6/7/6	Histopathology, follow-up imaging	0/178	0/178<br><br>Stage I, 0%<br>Stage II, NR<br>Stage III, NR	NR
Riedl, 2014 (Retro – IV)	134	PET/CT	Stage I to IIIC	- Stage I/IIA/IIB/IIIA/IIIB/IIIC, %: 15/33/35/10/6/1<br>- IDC/ILC/Mixed, %: 92/1/3<br>- ER+/HER2-, 56%<br>- HER2+, 19%<br>- TNBC, 21%	Histology	Unsuspected extra-axillary regional nodes were found in 11% (n=15) and distant mets in 15% (n=20), w 5% (n=7) demonstrating both<br><br>Distant mets: 16 osseous, 6 distant nodal, 5 liver, 2 lung, and 1 splenic; 7 pts ≥1 distant metastatic site.	N=20 upstaged:<br>- 5% Stage I<br>- 5% Stage IIA<br>- 17% Stage IIB<br>- 31% Stage IIIA<br>- 50% Stage IIIB<br>- 50% Stage IIIC.	NR

ABC, advanced breast cancer; ALND, axillary lymph node dissection; BC, breast cancer; BS, bone scan; C/A/P, chest/abdomen/pelvis; ce, contrast-enhanced; CI, confidence interval; CXR, chest X-Ray; DCIS, ductal carcinoma in situ; DWI, diffusion-weighted imaging; EBC, early breast cancer; IBC, inflammatory breast cancer; IDC, invasive ductal carcinoma; ILC, invasive lobular carcinoma; LABC, locally advanced breast cancer; LN, lymph node; NA, not applicable; NACT, neoadjuvant chemotherapy; NR, not reported; RCC, renal cell carcinoma; RR, relative risk; RT, radiation therapy; TNBC, triple negative breast cancer; US, ultrasound; WB, whole body.

Supplementary Table S3. Literature search strategy

**Clinical Practice Guidelines**				
**Strategy Type**	**Websites**	**Search**	**Date**	**Results**
Oncology-based websites	AHRQ Evidence Reports, American Society of Clinical Oncology (ASCO), British Columbia Cancer Agency (BCCA), Cancer Care Ontario (CCO), Cancer Council Australia, European Association of Nuclear Medicine (EANM), European Society of Medical Oncology (ESMO), National Comprehensive Cancer Network (NCCN), National Institute for Health and Care Excellence (NICE)	breast cancer	Apr 26, 2024	8
Guideline databases	ECRI	breast cancer	Apr 26, 2024	3/83
	TRIP Pro	breast cancer patients, computed tomography, positron emission tomography, magnetic resonance, upstaging from_date:2019 to_date:2024, guidelines	Apr 26, 2024	2/32
Biomedical literature databases	PubMed	("breast neoplasms"[MeSH Terms]) AND ((guideline[Filter]) AND (humans[Filter]) AND (2019/4/26:2024/4/26[pdat]) AND (english[Filter]) AND (alladult[Filter]))	Apr 26, 2024	0/91

*Excluded guidelines specifically addressing breast imaging during covid-19 era.

**White Literature**			
**Database**	**Search Strategy**	**Date**	**Results**
Medline	Re-ran search below to cover period “2023-current”	26-Apr-2024	1/74
Medline	exp Breast Neoplasms/dg [Diagnostic Imaging]	6-Nov-2023	17/637
	exp Bone Neoplasms/dg, sc [Diagnostic Imaging, Secondary]		
	exp Triple Negative Breast Neoplasms/dg [Diagnostic Imaging]		
	breast cancer.mp.		
	1 or 2 or 3 or 4		
	asymptomatic.mp.		
	newly diagnosed.mp.		
	6 or 7		

	5 and 8		
	magnetic resonance imaging.mp. or exp Magnetic Resonance Imaging/		
	MRI.mp.		
	exp Mass Chest X-Ray/		
	exp Tomography, X-Ray Computed/		
	bone scan.mp. or exp Radionuclide Imaging/		
	exp Radiography, Thoracic/ or thorax radiography.mp		
	positron emission tomography.mp. or exp Positron-Emission Tomography/		
	PET.mp.		
	CT.mp.		
	exp Fluorodeoxyglucose F18/		
	10 or 11 or 12 or 13 or 14 or 15 or 16 or 17 or 18 or 19		
	9 and 20		
	remove duplicates from 21		
	limit 22 to (english language and humans)		
	limit 23 to yr="2013 -Current"		

Supplementary Figure S1. PRISMA flow diagram

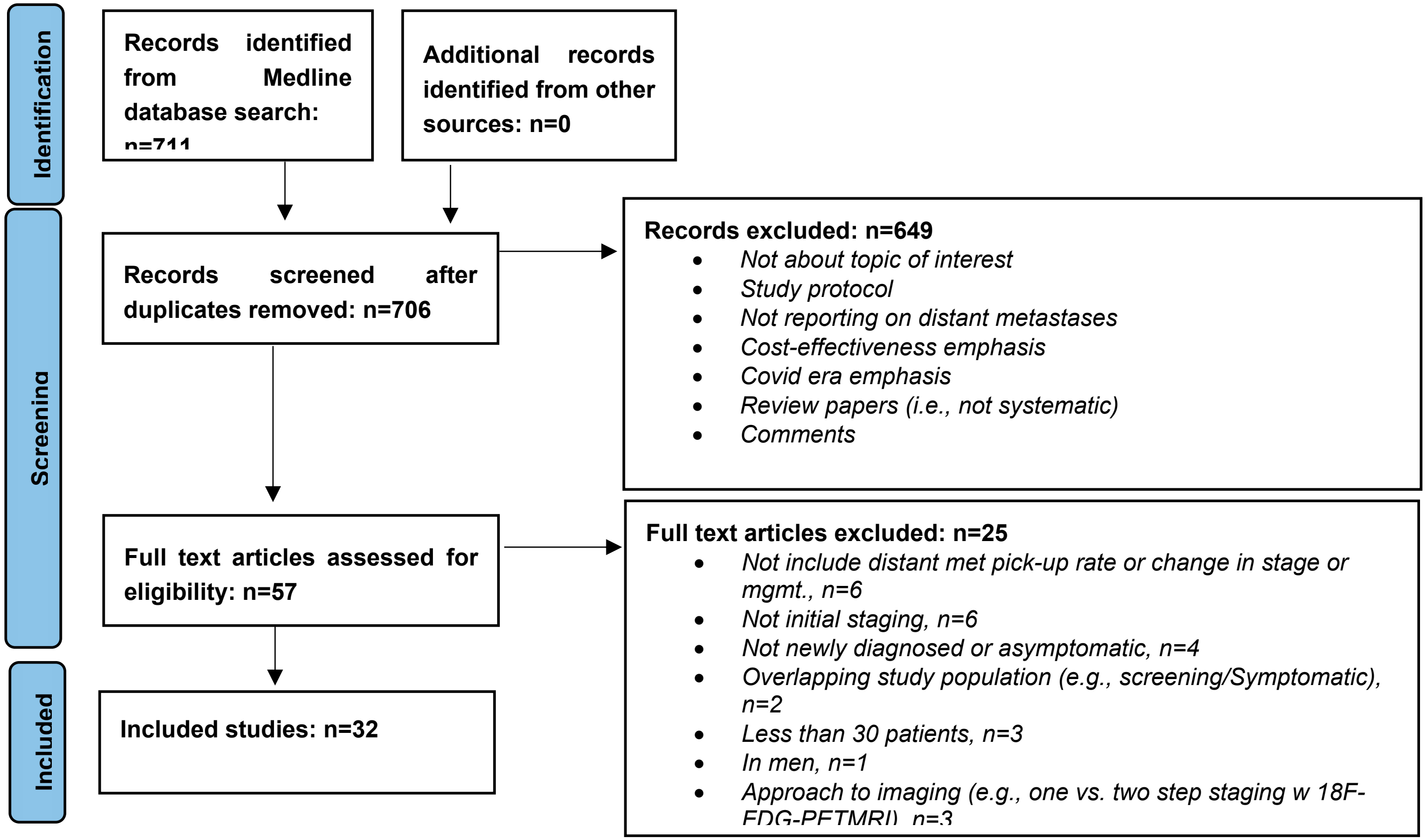

Adapted from: Page MJ, McKenzie JE, Bossuyt PM, Boutron I, Hoffmann TC, Mulrow CD, et al. The PRISMA 2020 statement: an updated guideline for reporting systematic reviews. BMJ 2021;372:n71. For more information, visit http://www.prisma-statement.org/

Supplementary Table S4. Pre-meeting survey and voting results

Survey Question	Voting (%)
Should AJCC 8th Edition Anatomic or Prognostic Stage Group be used when deciding about staging?	Anatomic: 60%<br>Prognostic: 40%<br>Abstain: 0%
Should staging recommendations be different based on post-operative versus neo-adjuvant setting?	Yes: 40%<br>No: 60%<br>Abstain: 0%
Should staging be modified by subtype in the neoadjuvant setting?	Agree: %<br>Agree with edits: 70%<br>Disagree: 0%
Should post-operative staging be modified by breast cancer subtype?	Yes: 40%<br>No: 60%<br>Abstain: 0%
Should localization clip be placed in the breast at time of biopsy/diagnosis?	Yes: 90%<br>No: 0%<br>Abstain: 10%
Should localization clip be placed in abnormal axillary node at time of biopsy/diagnosis?	Yes: 80%<br>No: 10%<br>Abstain: 10%
Should localization clip be placed in the breast primary prior to neoadjuvant chemotherapy (NAC)?	Yes: 80%<br>No: 10%<br>Abstain: 10%
Should localization clip be placed in the dominant positive axillary node prior to NAC?	Yes: 40%<br>No: 60%<br>Abstain: 0%
Stage 0-Is staging indicated for pure ductal carcinoma in situ (DCIS)?	Yes: 10%<br>No: 90%<br>Abstain: 0%
Stage IA/B- Is staging indicated?	Yes: 10%<br>No: 90%<br>Abstain: 0%
Stage IIA Anatomic (T0/1N1, T2N0)- Is staging indicated post-operatively?	Yes: 10%<br>Yes, but subtype specific: 30%<br>No: 60%<br>Abstain: 0%

Stage IIA Anatomic (T0/1N1, T2N0)- Is staging indicated prior to NAC?	Yes: 50%<br>Yes, but subtype specific: 20%<br>No: 30%<br>Abstain: 0%
Stage IIA Anatomic (T0/1N1, T2N0)- Do we need to split T0/1N1 and T2N0?	Yes: 40%<br>No: 50%<br>Abstain: 10%
Stage IIB Anatomic (T2N1, T3N0)- Is staging indicated post-operatively?	Yes: 100%<br>No: 0%<br>Abstain: 0%
Stage IIB Anatomic (T2N1, T3N0)- Is staging indicated prior to NAC?	Yes: 100%<br>No: 0%<br>Abstain: 0%
Stage IIB Anatomic (T2N1, T3N0)- Do we need to split T0/1N1 and T2N0?	Yes: 10%<br>No: 80%<br>Abstain: 10%
Stage III- Is staging indicated?	Yes: 100%<br>No: 0%<br>Abstain: 0%
Stage IV- Should we make a statement on frequency of scans?	Yes: 50%<br>No: 40%<br>Abstain: 10%
Is chest X-ray an adequate staging investigation?	Yes: 0%<br>No: 100%<br>Abstain: 0%
Is US of liver an adequate staging investigation?	Yes: 40%<br>No: 60%<br>Abstain: 0%
When requesting CT based imaging, which test should be ordered at baseline?	CT-Chest/Abdomen: 30%<br>CT-Chest/Abdomen±Pelvis: 20%<br>CT-Chest/Abdomen/Pelvis: 50%<br>Abstain: 0%
Is a bone scan an adequate staging investigation?	Yes: 100%<br>No: 0%

	Abstain: 0%
When staging is indicated, should PET scan be ordered routinely?	Yes: 40%<br>No: 60%<br>Abstain: 0%
Should PET be ordered based on specific breast cancer subtypes?	Yes: 30%<br>No: 60%<br>Abstain: 10%
Should PET be ordered for inflammatory breast cancer?	Yes: 90%<br>No: 10%<br>Abstain: 0%
Should PET be ordered for lobular breast cancer?	Yes: 20%<br>No: 80%<br>Abstain: 0%
Should MR of Breast/Chest Wall be used routinely during work up of breast cancer?	Yes: 10%<br>No: 90%<br>Abstain: 0%
Should MR of Breast/Chest Wall be standard for invasive lobular carcinoma?	Yes: 50%<br>No: 50%<br>Abstain: 0%
Should MR Breast/Chest Wall be standard for BRCA1/2+ patients?	Yes: 80%<br>No: 20%<br>Abstain: 0%
Should CT Head or MR Brain be part of staging for all-comers?	Yes: 0%<br>No: 100%<br>Abstain: 0%
Should CT Head or MR Brain be part of staging for HER2+ patients?	Yes: 30%<br>No: 70%<br>Abstain: 0%
Should CT Head or MR Brain be part of staging for TNBC patients?	Yes: 0%<br>No: 90%<br>Abstain: 10%
Should molecular breast imaging (MBI) be considered?	Yes: 0%<br>No: 70%

	Abstain: 30%

Supplementary Table S5. Expanded polling results for recommendations.

Recommendation	Voting (%)	Comments for disagreement
For patients newly diagnosed with breast cancer and without signs and/or symptoms of distant disease, the decision to order staging investigations should be based on the Anatomic Stage Group as per the 8th Edition American Joint Committee on Cancer (AJCC) TNM Staging System.	Agree: 94%<br>Agree with edits: 6%<br>Disagree: 0%	NA
The decision to order staging investigations should not differ based on adjuvant or neoadjuvant approach nor should it be modified by subtype in either the neoadjuvant or post-operative settings.	Agree: 75%<br>Agree with edits: 6%<br>Disagree: 19%	•PET or ultrasound of axilla for clinically node negative should be warranted before neoadjuvant treatment<br>•High risk cancers (i.e. triple neg) are more likely to present at diagnosis with visceral metastases than low grade<br>•The consequence of finding distant disease is very different depending on the circumstances
•Imaging for local and regional work-up with bilateral mammography as well as breast and axillary ultrasound is indicated for all patients with suspected breast cancer.<br>•For locally advanced disease (Stages IIB-IIIC), comprehensive imaging, including mammography, ultrasound, +/- MRI, is indicated to evaluate the extent of local and regional disease.<br>•Preoperative breast MRI should be considered for patients diagnosed with breast cancer where additional information regarding disease extent could influence treatment decisions.<br>•Decision to conduct MRI should be made in consultation with the patient, considering the balance of benefits and risks and patient preferences.<br>•In patients diagnosed with invasive lobular carcinoma for whom additional information about disease extent could influence treatment<br>•Preoperative breast MRI is recommended in the following situations, based on the opinion of the Working Group:<br>•To aid in surgical planning for breast-conserving surgery in patients with suspected or known multicentric or multifocal disease.<br>•To identify additional lesions in patients with dense breasts.<br>•To determine the presence of pectoralis major muscle/chest wall invasion in patients with posteriorly located tumours or when invasion is suspected.<br>•To aid in surgical planning for skin/nipple-sparing mastectomies or for procedures like autologous reconstruction, oncoplastic surgery, and breast-conserving surgery with suspected nipple/areolar involvement.<br>•For patients with familial/hereditary breast cancer who have not had recent breast MRI as part of screening or diagnosis.<br>•For patients who have had mantle radiation.	Agree: 87%<br>Agree with edits: 13%<br>Disagree: 0%	NA
The placement of a marker is indicated at the time of core needle biopsy to mark the location of the primary tumour(s) and axillary node(s), especially if neoadjuvant therapy is planned. The decision to place markers for axillary nodes should be made in consultation with the breast surgeon and in accordance with local institutional practices.	Agree: 94%<br>Agree with edits: 6%<br>Disagree: 0%	NA

For breast conserving surgery and radiation therapy planning, clips should be placed in the surgical bed, especially if oncoplastic surgery is performed. This is useful to guide radiotherapy planning of boosts and allows for the option of accelerated partial breast irradiation.		
Routine chest X-rays are not indicated for staging but may be used for initial evaluation in patients with respiratory symptoms or for baseline assessment in certain clinical scenarios.	Agree: 94%<br>Agree with edits: 6%<br>Disagree: 0%	•If symptoms are apparent, CT can be favored for staging rather than a chest-Xray
CT Thorax/Abdomen ± Pelvis is indicated for patients with Stages IIB-IIIC. Routine use in early-stage disease (Stages 0-IIA) is not recommended.	Agree: 100%<br>Agree with edits: 0%<br>Disagree: 0%	NA
Bone Scan is indicated for patients with Stages IIB-IIIC. Routine use in early-stage disease (Stages 0-IIA) is not recommended.	Agree: 87%<br>Agree with edits: 13%<br>Disagree: 0%	NA
Ultrasound of the liver is a reasonable alternative to CT abdomen if CT is contraindicated or not available.	Agree: 81%<br>Agree with edits: 6%<br>Disagree: 13%	•CT should be the preferred alternative<br>•Not for stage III disease where pre-test probability is higher
PET-CT is an alternative, but not an additional staging investigation, to conventional imaging with computed tomography and bone scan in patients with Stage IIB-IIIC presentation	Agree: 100%<br>Agree with edits: 0%<br>Disagree: 0%	NA
PET-CT is not currently indicated for lobular breast cancer (due to low grade activity but is under further investigation)	Agree: 93%<br>Agree with edits: 0%<br>Disagree: 7%	•Only 30% of lobular breast cancers are not FDG-avid – so if PET is negative then patient can move ahead with routine CT and bone scan staging
PET-CT may be recommended for patients with inflammatory breast cancer	Agree: 93%<br>Agree with edits: 0%<br>Disagree: 7%	•Lack of convincing evidence to support
PET-CT may be useful when conventional imaging results in equivocal findings	Agree: 100%<br>Agree with edits: 0%<br>Disagree: 0%	NA
CT-head or MR-brain is not routinely indicated for staging asymptomatic patients, irrespective of sub-type (i.e., HER2-positive and TNBC).	Agree: 81%<br>Agree with edits: 19%<br>Disagree: 0%	NA
Imaging workup should be completed in a timeframe that will not impede the initiation or continuation of treatment(s). Timeframe should be guided by the Canadian Society of Breast Imaging (CSBI) wait time benchmarks.	Agree: 100%<br>Agree with edits: 0%<br>Disagree: 0%	NA
Advanced PET technology, focusing on distinct metabolic processes in breast cancer imaging, demonstrates promise but is not currently routine standard of care. Reassessing and disseminating knowledge on these and other emerging technologies as evidence evolves is crucial to enhancing diagnostic accuracy and providing state-of-the-art care for breast cancer patients in Canada.	Agree: 87%<br>Agree with edits: 13%<br>Disagree: 0%	NA
